# Genetic Testing After Heart Transplantation Uncovers Heritable Disease and Drives Family Screening

**DOI:** 10.1016/j.jacadv.2026.103043

**Published:** 2026-07-31

**Authors:** Carlos Moliner-Abós, Diego Cabrera Argaña, David Belmar Cliville, Sonia Rivas, Maria Generosa Crespo-Leiro, Adrian Peña Hidalgo, Ramón Garrido Gonzalez, Pablo Martín Marín, Miriam Gómez Molina, Rebeca Lorca, Aridane Cárdenes León, Francisco González Vilchez, Juan José Rodriguez Arias, Mayte Basurte Elorz, María Valverde, Jose Maria Larrañaga-Moreira, Milena Antunez-Ballesteros, Fernando de Frutos, Mercedes Rivas-Lasarte, José Manuel Sobrino Márquez, Iris Paula Garrido Bravo, Beatriz Diaz Molina, Carmen Acosta Calero, Cristina Goena-Vives, Ana García-Álvarez, Silvia Vilches, Elena Garcia Romero, Manuel Gomez Bueno, Jose Manuel Garcia Pinilla, Vanesa Alonso Fernandez, Juan Pablo Ochoa, Marta de Antonio-Ferrer, Benjamin Rodriguez-Santiago, Sonia Mirabet-Pérez

**Affiliations:** a Cardiology Department, Hospital de la Santa Creu i Sant Pau, Barcelona, Spain; b Cardiology Department, Hospital Universitari de Girona Dr. Josep Trueta, Girona, Spain; c IR SANT PAU, Barcelona, Spain; d Girona Biomedical Research Institute (IDIBGI), Girona, Spain; e Centro de Investigación Biomédica en Red de Enfermedades Cardiovasculares (CIBERCV), Madrid, Spain; f Universitat Autònoma de Barcelona (UAB), Barcelona, Spain; g Health in Code, A Coruña, Spain; h Cardiology Department, Hospital Universitario 12 de Octubre, Madrid, Spain; i Instituto de Investigación Sanitaria Hospital 12 de Octubre (imas12), Madrid, Spain; j Cardiology Department, Complexo Hospitalario Universitario A Coruña (CHUAC), INIBIC, CIBERCV, A Coruña, Spain; k Universidade da Coruña (UDC), A Coruña, Spain; l Advanced Heart Failure and Transplantation Unit, Cardiology Department, Hospital Universitari de Bellvitge, L’Hospitalet de Llobregat, Barcelona, Spain; m Cardiology Department, Hospital Universitario Puerta de Hierro Majadahonda, Madrid, Spain; n Cardiovascular Imaging and Inherited Cardiac Diseases Unit, Cardiology Department, Hospital Universitario Virgen del Rocío, Sevilla, Spain; o Cardiology Department, Hospital Universitario Virgen de la Arrixaca, Murcia, Spain; p Inherited Cardiac Diseases Unit, Área del Corazón, Hospital Universitario Central de Asturias (HUCA), Oviedo, Spain; q Departamento de Medicina, Universidad de Oviedo, Oviedo, Spain; r Instituto de Investigación Sanitaria del Principado de Asturias (ISPA), Oviedo, Spain; s Redes de Investigación Cooperativa Orientadas a Resultados en Salud (RICORs), Madrid, Spain; t Cardiology Department, Hospital Universitario de Gran Canaria Doctor Negrín, Las Palmas de Gran Canaria, Spain; u Cardiology Department, Hospital Universitario Marqués de Valdecilla, Santander, Spain; v Cardiology Department, Hospital Clínic, IDIBAPS, University of Barcelona, Barcelona, Spain; w Cardiology Department, Clínica Universidad de Navarra, Pamplona, Spain; x Bioheart Group, Bellvitge Biomedical Research Institute (IDIBELL), L’Hospitalet de Llobregat, Barcelona, Spain; y Heart Failure and Inherited Cardiac Diseases Unit, Department of Cardiology, Donostia University Hospital, BioGipuzkoa Health Research Institute, Donostia, Spain; z Inherited Cardiomyopathies Group, CNIC (Centro Nacional de Investigaciones Cardiovasculares), Madrid, Spain; aa European Reference Network for Rare and Low Prevalence Complex Diseases of the Heart (ERN GUARD-Heart), Amsterdam, the Netherlands; ab Instituto de Salud Carlos III, Madrid, Spain; ac Department of Clinical Sciences, School of Medicine, University of Barcelona, Barcelona, Spain; ad Heart Failure and Inherited Cardiac Diseases Unit, Cardiology Department, Hospital Universitario Virgen de la Victoria, IBIMA, Málaga, Spain; ae Departamento de Medicina y Dermatología, Universidad de Málaga, Málaga, Spain; af Servei de Genètica, Hospital de Sant Pau, Barcelona, Spain; ag Centro de Investigación Biomédica en Red de Enfermedades Raras, Madrid, Spain; ah Unitat Mixta de Recerca en Medicina Genòmica, UAB-IR SANT PAU, Barcelona, Spain

**Keywords:** cardiomyopathy, etiologic reclassification, family screening, genetic testing, heart transplantation

## Abstract

**Background:**

Genetic testing (GT) is established in ambulatory cardiomyopathy (CM), but its utility after heart transplantation (HTx) recipients remains poorly characterized.

**Objectives:**

This study aimed to evaluate the clinical utility of GT in adult HTx recipients with CM, focusing on etiologic reclassification, family cascade screening, and the genetic architecture of end-stage disease.

**Methods:**

GenbaseHTx is a nationwide, multicenter retrospective study of adult HTx recipients transplanted for CM across 12 Spanish centers (2010-2023). GT results were centrally adjudicated using American College of Medical Genetics and Genomics criteria. Outcomes included prevalence of pathogenic/likely pathogenic variants, etiologic reclassification after GT, and cascade screening activation.

**Results:**

Among 657 HTx recipients, a pathogenic/likely pathogenic variant was identified in 53% (351/657). GT led to etiologic reclassification in 35% (231/657) (42% when performed post-HTx −106/253-). Family screening (performed in 69% of families −224/328-) identified affected relatives in 22% (19/90) of genotype-negative and 42% (49/107) of genotype-positive cases. Notably, a genetic etiology was identified in 36% (15/42) of CM initially attributed to acquired or “second-hit” causes. In dilated cardiomyopathy, the genetic architecture of transplanted patients differed from ambulatory cohorts, with lower *TTN* variant prevalence and enrichment of arrhythmogenic genes.

**Conclusions:**

GT remains clinically actionable after HTx, enabling etiologic reclassification and driving cascade screening. These findings support systematic GT in HTx recipients with CM, including those with prior environmental triggers or second-hit etiologies, and regardless of time from transplantation.

Cardiomyopathies (CMs) represent the leading indication for heart transplantation (HTx) worldwide and account for more than half of adult transplant procedures.^1^ Over the past decade, genetic testing (GT) has become a central component of the diagnostic evaluation of CM, with current guidelines recommending its use across all major phenotypes to refine diagnosis, inform prognosis, and guide family management. However, the clinical utility of GT has been primarily established in ambulatory patients with stable or moderately advanced disease.^2^^,^^3^

Patients who progress to end-stage heart failure (ESHF) requiring HTx represent the extreme end of the CM spectrum, characterized by rapid disease progression and refractoriness to guideline-directed therapies.^4^ In this setting, etiologic investigation is often deprioritized once transplantation is achieved, under the implicit assumption that defining the genetic basis of CM after HTx has limited clinical relevance for the recipient. Whether this assumption is justified remains largely unexplored.

Importantly, HTx does not eliminate the clinical consequences of an underlying heritable CM. Genetic diagnosis may enable etiologic reclassification, challenge the attribution of disease to purely acquired or “second-hit” causes, and—critically—guide cascade screening and preventive care in at-risk relatives. Despite these potential implications, systematic data on the yield and clinical impact of GT performed after HTx are scarce, and existing studies^5^, ^6^, ^7^, ^8^ are limited by small sample size, heterogeneous testing strategies, and lack of standardized variant adjudication.

We therefore designed the GenbaseHTx study to evaluate the clinical utility of GT in a large, nationwide cohort of adult HTx recipients with CM. We hypothesized that GT, even when performed years after transplantation, would 1) identify a substantial burden of heritable CM, 2) lead to etiologic reclassification in a significant proportion of patients, 3) trigger clinically actionable family screening, and 4) that the genetic architecture of CMs progressing to transplantation differs from that of ambulatory disease, suggesting distinct determinants of rapid progression.

## Methods

### Study design and population

The GenbaseHTx study is a retrospective, multicenter study of adult HTx recipients conducted across 12 Spanish transplant centers. We screened all consecutive adult patients (>18 years) who underwent HTx between January 1, 2010, and December 31, 2023. For the primary analysis (diagnostic yield, reclassification), we included all patients transplanted for any CM phenotype. For secondary analyses (family screening), we included a subset of probands who had undergone comprehensive, contemporary next-generation sequencing (NGS)–based GT (PROBANDS & NGS COHORT). A detailed flowchart of patient selection is provided in Supplemental Figure 1.

The study was approved by the institutional ethics committees of all participating centers. Due to its retrospective nature, the requirement for informed consent was waived.

### Data sources and clinical variables

Clinical data were obtained from the Spanish Heart Transplantation Registry (RNTC, the acronym corresponding to the Spanish name Registro Nacional de Trasplante Cardíaco),^9^ a nationwide voluntary registry that encompasses all heart transplant procedures conducted in Spain since 1984. The RNTC collects standardized variables, including demographic and clinical characteristics of recipients at the time of listing. This registry has been continually active for over 30 years and represents the totality of transplant activity in the country.

Diagnoses are coded at the time of listing using a standardized catalog of diagnostic categories. Patients were eligible for inclusion if they were assigned a diagnostic code corresponding to dilated CM (DCM), arrhythmogenic CM (ACM), restrictive CM (RCM), or hypertrophic CM (HCM). Cases labeled as “other cardiac disease” were reviewed individually and included only if no alternative etiology was identified. Codes for myocarditis and anthracycline cardiotoxicity were also eligible, but GT was not systematically performed in these categories due to center-specific practices: either a clearly established inflammatory etiology (virology/histology) or lack of perceived clinical indication for GT. Patients with a primary diagnosis of valvular heart disease were excluded. However, those with secondary functional valvular disease in the context of an established CM were included.

Data were supplemented by detailed retrospective review of medical records, including clinical history, imaging, and GT reports, at each participating center.

### Phenotypical and etiological classification

Given that the classification of CM evolved over the study period, CM phenotypes were generally retained as originally recorded in the RNTC database at inclusion, encompassing DCM, HCM, and RCM. Phenotypic information was only reviewed in cases of missing data or when discrepancies were noted with other available clinical information, in which case the phenotype was updated accordingly.

Initial (pre-GT) etiology was adjudicated based on all information available at the time of HTx listing, retrieved from the RNTC and medical records. We applied the following definitions:

• Idiopathic CM: Absence of an identifiable secondary cause and no known family history of CM or sudden cardiac death (SCD) before age 35.
• Familial CM: Presence of at least one first- or second-degree relative with CM or premature SCD.
• *Secondary or “Second-Hit” CM:* CM occurring in the presence of a recognized clinical or environmental stressor associated with myocardial dysfunction (chemotherapy exposure, alcohol abuse, peripartum state, myocarditis, or tachyarrhythmia-induced CM). This was considered a distinct etiologic category and was not included within idiopathic CM.

For the purpose of this study, we defined etiologic reclassification as any change from the pretest etiologic category to a different post-test category based on genetic findings. This definition encompasses both true diagnostic reclassification (change in the clinical entity, eg, from myocarditis to genetic CM) and etiologic specification (identification of a genetic cause without changing the phenotypic entity, eg, from idiopathic DCM to DCM due to TTN).

### Genetic testing and variant interpretation

GT was part of routine clinical practice at participating centers. For patients transplanted prior to the widespread availability of genomics, GT was performed during long-term follow-up to enable family screening (FS) and definitive etiologic diagnosis, reflecting the evolving standards of care. GT was performed at each participating center according to local clinical practice and using their respective reference laboratories. Participating centers were asked to provide detailed information on the type of GT (Sanger/NGS-gene panel/NGS-exome/NGS-genome/other), number and identity of genes included in each genetic analysis. Specific attention was given to the inclusion of filamin C (FLNC), given that its association with DCM was first established during the study period.^10^ Copy-number variant (CNV) analysis and structural variant detection were performed when included in the NGS-based workflows of the participating laboratories. Patients in which technical information about GT was not available were assumed to have undergone NGS-based testing.

All GT results were centrally reviewed by a cardiologist with expertise in cardiovascular genetics (D.C.A.) using the classification framework proposed by the American College of Medical Genetics and Genomics (ACMG).^11^ In cases of discrepancy between the local interpretation and centralized review, the corresponding center was contacted to provide additional evidence supporting the original variant classification.

Variants were categorized as pathogenic (P), likely pathogenic (LP), variant of uncertain significance, likely benign, or benign, following the 2015 ACMG–Association for Molecular Pathology guidelines.^11^ Positive GT was considered when an LP/P variant was found. When available, gene-specific modifications to ACMG criteria (as proposed by ClinGen and related expert panels) were applied to ensure accurate classification. The detailed criteria supporting final classification for each variant are provided in Supplemental Table 1.

### Genotype-phenotype correlation analysis

For downstream genotype-phenotype correlation analyses, genes harboring P/LP variants were assigned to predefined functional groups based on protein function, biological pathway, and subcellular localization. To ensure a homogeneous study population, this analysis was restricted to patients with a primary DCM phenotype. Specifically, metabolic and infiltrative phenocopies—including those caused by variants in syndromic glycogenosis, Danon disease, and mitochondrial DNA—were excluded. Patients with Duchenne muscular dystrophy-related DCM were also analyzed separately from the main functional groups given its distinct clinical course.

Classification was informed by consolidated evidence from published literature and biological databases.^12^ The final functional groups were: 1) structural cytoskeleton/Z-disk; 2) desmosomal; 3) nuclear envelope; 4) motor sarcomeric; 5) *TTN*; 6) regulatory pathways; and 7) other genes. For clinical risk stratification, a high-risk arrhythmogenic profile was further defined by grouping variants in genes known for high electrical instability and rapid progression, specifically including desmosomal genes (*DSP, PKP2, DSG2, DSC2, JUP*), nuclear envelope components (*LMNA, EMD, TMEM43*), and specific pro-arrhythmic drivers (*FLNC, PLN, RBM20*). The inclusion of *EMD* within this group is justified by its shared role in nuclear envelope stability and its clinical presentation characterized by severe conduction disease and high arrhythmic burden, matching the phenotypic profile of *LMNA* carriers in our cohort.

Given its unique prevalence and distinct clinical behavior, *TTN* was analyzed as a standalone category. Patients with more than one P/LP variant were excluded from these analyses to ensure a clear genotype-phenotype attribution. Furthermore, patients with a negative genetic study were included as the reference group for clinical outcome comparisons, with age at heart transplantation used as the time scale for all survival analyses.

### Family evaluation

All participating centers had established inherited cardiovascular disease units and followed guideline recommendations for the evaluation of familial CM.^2^^,^^13^ Moreover, clinicians were encouraged to construct a three-generation pedigree and to initiate cascade genetic screening in relatives when an LP/P variant was identified. In the presence of a variant of unknown significance in a CM-curated gene, co-segregation analysis was also encouraged when feasible.

As part of the study, information on family history of CM, SCD, or skeletal myopathy was systematically collected for each proband to support variant interpretation and to assess the potential clinical impact of molecular diagnosis in at-risk family members.

### Statistical analysis

Continuous variables are expressed as mean ± SD or as median (IQR), as appropriate, based on data distribution. Groups were compared using Student’s *t*-test or the Mann-Whitney *U* test for 2-group comparisons and analysis of variance or the Kruskal-Wallis test for comparisons of more than 2 groups. Categorical variables are expressed as counts (percentages) and were compared using the chi-square or Fisher exact test, as appropriate.

Genetic variant frequencies across CM phenotypes and in comparisons with external cohorts were considered exploratory. No statistical adjustment for multiple testing was applied to these comparisons, and findings should be considered exploratory and hypothesis-generating.

For the analysis of time from birth to HTx, the transplantation-free survival according to age was estimated using the Kaplan-Meier method, and groups were compared with the log-rank test. All survival models used age at transplantation as the time scale to account for the lifelong risk associated with germline variants, with genotype-negative patients serving as the reference group. To assess the association of specific genetic variant groups with the rate of progression to transplantation, we fitted Cox proportional hazards models. To account for potential between-center heterogeneity, we used a shared frailty term for transplant center. To address intercohort heterogeneity due to changes in clinical practice and GT availability over the 13-year study period, we defined 3 temporal cohorts based on year of transplantation (2010-2014, 2015-2019, and 2020-2023) and included them as fixed covariates. The proportional hazards assumption was tested using Schoenfeld residuals. Patients were categorized using 2 complementary approaches: first, by predefined functional gene groups based on established biological pathways; and second, by a clinical risk-based stratification comparing an arrhythmogenic-high-risk cluster against other genotypes. HRs with 95% CIs were derived from the models. As a sensitivity analysis, we additionally fitted accelerated failure time models with log-normal and Weibull distributions, including the same covariates and shared frailty by hospital.

For all survival analyses, a complete-case analysis approach was used. Missing data were minimal (<1% for covariates, 0.2% for functional gene group, and 0% for clinical risk group), and Little's MCAR test was not significant (*P* = 0.914) (Supplemental Table 2).

Trends across ordered temporal cohorts were assessed using the Cuzick nonparametric test for trend. A 2-sided *P* value <0.05 was considered statistically significant for primary comparisons. All analyses were performed using Stata version 15 (StataCorp).

## Results

### Study population

Between 2010 and 2023, 3,982 adult HTx were performed in Spain, of which 2,583 occurred at the 12 centers participating in the GenbaseHTx study. After excluding non-CM etiologies (n = 1,268), patients who died without a contemporary GT (n = 188), those in whom GT was not performed (n = 172), and those with an alternative etiology identified after retrospective review (n = 106), the final study cohort comprised 657 adult HTx recipients with CM. This represents 56% of all CM-related transplant procedures performed at participating centers during the study period (Supplemental Figure 1). A comparison of baseline characteristics between the included and excluded patients is available in Supplemental Table 3.

The median age at HTx was 53.0 years (IQR: 43.6-60.6); 67% were male, and DCM was the most common indication for HTx (68%), followed by HCM (21%), ACM (6%), and RCM (5%). Most patients were probands (90%), and 34% underwent urgent transplantation. Baseline clinical characteristics are summarized in Table 1.

**Table 1:** Baseline Characteristics of the Genbase-Htx Study Cohort, by Cardiomyopathy Phenotype

Variable	DCM (n = 448)	HCM (n = 136)	ACM (n = 39)	RCM (n = 34)	Total (N = 657)	*P* Value
Age, y	53.0 [43.2–60.7]	53.1 [44.7–60.9]	53.0 [44.7–57.1]	52.7 [39.9–61.1]	53.0 [43.6–60.6]	0.91
Male, n (%)	328 (74)	74 (54)	25 (64)	13 (42)	440 (67)	<0.01
Proband, n (%)	412 (92)	123 (90)	39 (100)	23 (68)	594 (90)	<0.01
Family history CM or SD, n (%)	113 (33)	44 (45)	12 (39)	8 (53)	177 (36)	0.08
Skeletal myopathy, n (%)	15 (5)	2 (2)	2 (15)	1 (3)	20 (4)	0.15
Major ventricular arrhythmias, n (%)	100 (31)	18 (19)	20 (63)	2 (15)	140 (30)	<0.01
Cardiogenic shock, n (%)	106 (33)	12 (13)	5 (16)	0 (0)	123 (26)	<0.01
LVEF, %	22 [18–28]	37 [26–50]	33 [25–50]	44 [38–55]	25 [20–33]	<0.01
LVEDD, mm	66 [59–72]	51 [45–59]	54 [47–66]	48 [42–55]	63 [53–70]	<0.01
TAPSE, mm	15 [12–18]	15 [13–17]	11 [9–13]	11 [9–13]	15 [12–17]	<0.01
ICD, n (%)	362 (82)	106 (78)	38 (97)	7 (23)	513 (79)	<0.01
CRT, n (%)	286 (65)	111 (83)	35 (90)	22 (76)	453 (71)	<0.01
Urgent listing, n (%)	173 (38)	28 (20)	12 (31)	7 (23)	220 (34)	<0.01
Ventricular assist device, n (%)	96 (21)	13 (9)	6 (15)	2 (6)	117 (18)	<0.01

Characteristics of the 659 heart transplant recipients included in the Genbase-Htx genetic study. Data are presented as n (%) for categorical variables and median [IQR] for continuous variables.

ACM = arrhythmogenic cardiomyopathy; DCM = dilated cardiomyopathy; HCM = hypertrophic cardiomyopathy; RCM = restrictive cardiomyopathy; CM = cardiomyopathy; CRT = cardiac resynchronization therapy; ICD = implantable cardioverter-defibrillator; LVEDD = left ventricular end-diastolic diameter; LVEF = left ventricular ejection fraction; SD = sudden death; TAPSE = tricuspid annular plane systolic excursion.

GT was performed after HTx in 54% of patients (253/467 with available GT timing data), with a median interval from HTx to GT of 4.3 years (IQR: 1.5-7.6). In the remaining 46%, GT was performed prior to HTx (median interval 1.7 years, IQR: 0.7-3.5).

The proportion of patients undergoing pretransplant GT increased markedly over the study period, from 12% (13/106) in 2010 to 2014 to 41% (79/191) in 2015 to 2019, reaching 68% (124/177) in 2020 to 2023 (*P* for trend <0.001) (Supplemental Figure 2). This progressive integration of genetics into pretransplant evaluation reflects increased awareness and evolving clinical practice over the 13-year study period.

### Genetic testing reveals a high burden of heritable cardiomyopathy

GT was performed using NGS–based approaches, comprising targeted gene panels (78%) and whole-exome sequencing (7%). Among NGS-based studies, the median number of genes analyzed per test was 98 (IQR: 71-121). Sanger sequencing was in 12% and was used for familial cascade testing or for direct sequencing of transthyretin gene in cardiac amyloidosis. A P/LP variant was identified in 350 patients (53%, 95% CI: 49%-57%) in the overall cohort. Baseline characteristics of GT-positive compared with GT-negative patients are shown in Supplemental Table 4. In probands who underwent contemporary NGS-based GT, the diagnostic yield was 48%.

Diagnostic yield varied according to CM phenotype, reaching 46% (95% CI: 42%-51%) in DCM, 68% (95% CI: 60%-76%) in HCM, 56% (95% CI: 41%-71%) in ACM, and 71% (95% CI: 53%-84%) in RCM (Supplemental Table 5). These findings demonstrate a substantial burden of heritable disease among patients progressing to ESHF requiring HTx. Variant-level details, including ACMG classification and gene-specific distributions, are provided in Supplemental Table 1. Among HTx recipients with clinically evident skeletal myopathy (n = 21), the diagnostic yield of GT was higher than in unaffected patients (67% vs 50%, *P* = 0.13). Genes implicated in these patients are listed in Supplemental Table 6.

### Genetic testing leads to etiologic reclassification after heart transplantation

**Figure 1:**
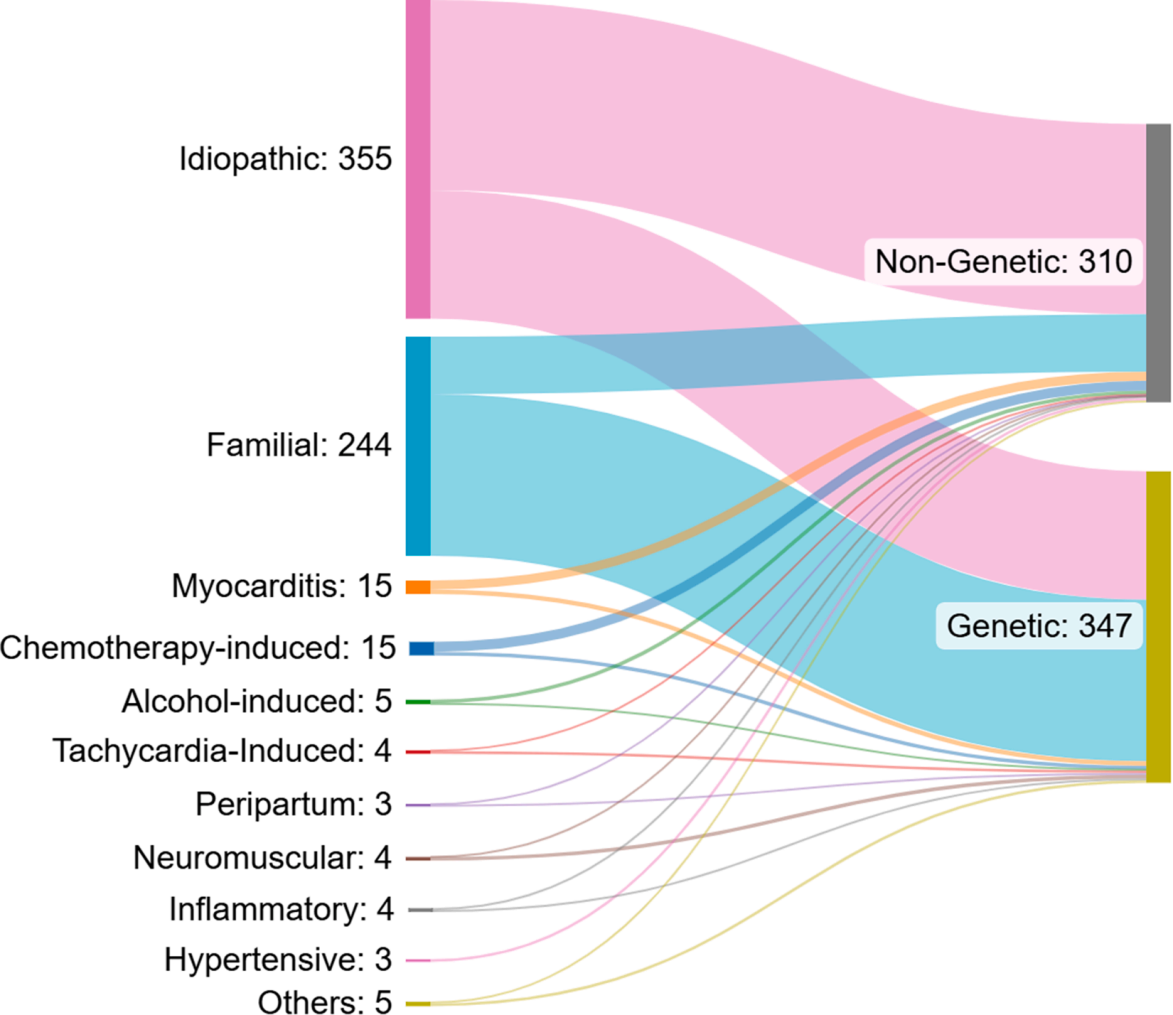
Etiologic Reclassification After Genetic Testing Sankey diagram showing reclassification of clinical etiology in 657 heart transplant recipients with cardiomyopathy. The left column shows initial clinical etiology (46% had prior genetic evaluation). The right column shows final classification after genetic testing (genetic = P/LP variant; nongenetic = no P/LP variant). Genetic testing reclassified 40% (143/355) of idiopathic cases and 36% (15/42) of presumed acquired or “second-hit” etiologies (eg, myocarditis, chemotherapy-induced) to a genetic cause. P/LP = pathogenic/likely pathogenic.

GT resulted in etiologic reclassification in 231 patients (35% of the overall cohort; 95% CI: 32%-39%). Reclassification most frequently involved reassignment from idiopathic to genetic CM (n = 143/355, 41%; 95% CI: 35%-46%). In 64/244 patients (26%; 95% CI: 21%-32%) GT did not identify a monogenic cause in families with clinically suspected familial and CM reclassification involved a change from familial CM to a nongenetic etiology (Figure 1). This finding should be interpreted with caution: in some cases, the initial “familial” label was retained from the national transplant registry without independent verification of family history; in others, the reported family history could not be confirmed as true CM or SCD due to lack of medical records or autopsy data—a common limitation in real-world clinical practice. Importantly, a negative genetic test in a family with suspected aggregation does not exclude heritable risk, as current genomic knowledge is incomplete and our analysis focused on monogenic causes. Therefore, clinical surveillance of at-risk relatives remains essential regardless of genetic results, and these families should not be considered nongenetic.

Among patients who underwent GT after HTx, etiologic reclassification occurred in 42%, despite a median interval of more than 4 years between HTx and GT. The full spectrum of etiologic transitions before and after GT is depicted in Figure 1 and Supplemental Table 7. These findings indicate that GT remains clinically informative even when performed years after HTx.

### Genetic testing activates family cascade screening

FS data were available for 328 probands (56% of the probands). Of these, 224 (68%) had documented FS, while in 104 (32%) no screening was performed (not offered or declined). Notably, cascade FS was not pursued more frequently in GT-negative probands than in GT-positive probands (41% [70/171; 95% CI: 34%-49%] vs 21% [34/157; 95% CI: 16%-28%]; *P* < 0.01).

**Figure 2:**
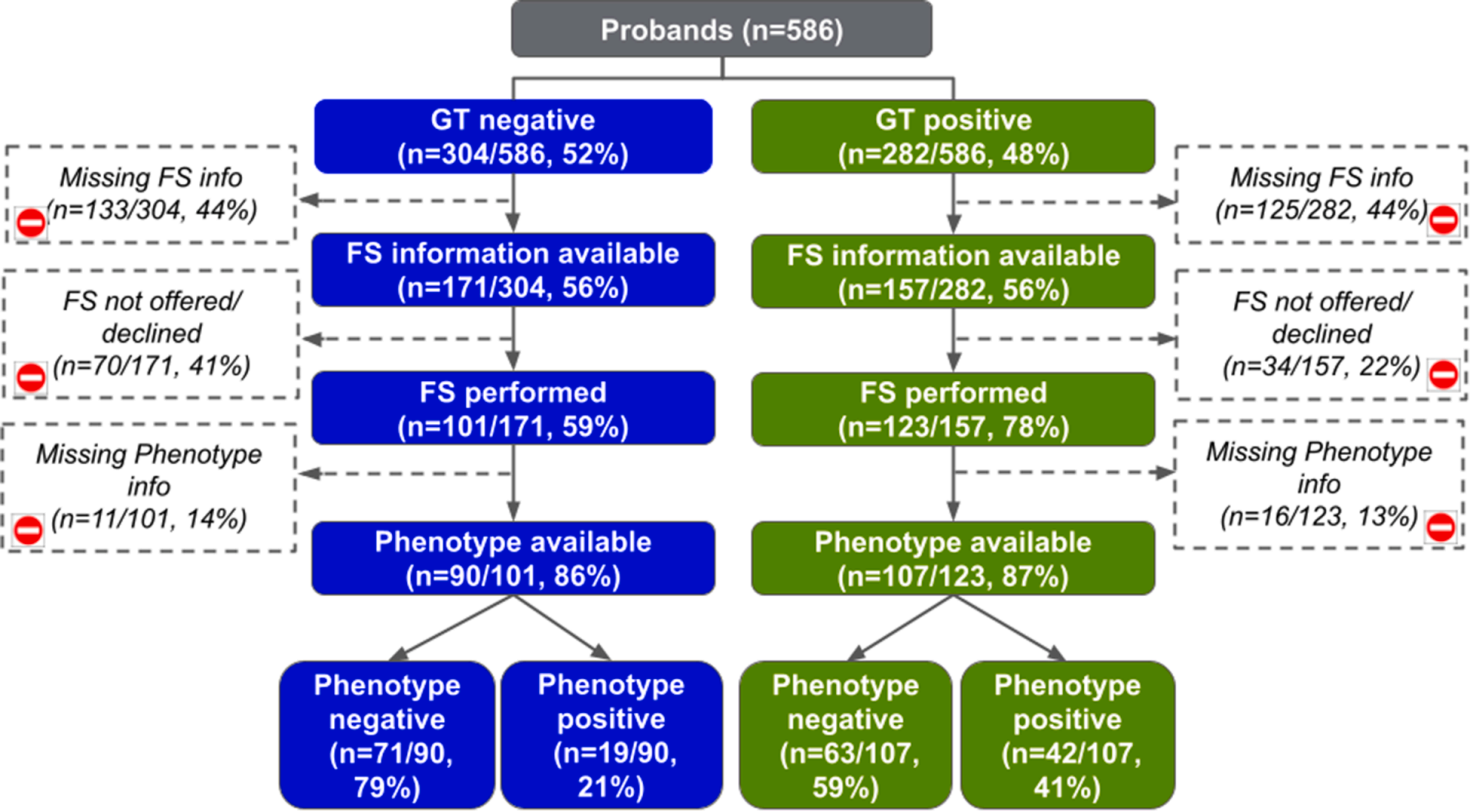
Family Cascade Screening by Proband Genotype Flow diagram of clinical evaluation in 586 families according to proband genetic testing status. Solid vertical arrows indicate proband families who continue to the next step of screening. Dashed horizontal lines indicate probands who are excluded from further analysis at that step, with the reason for exclusion stated. GT = genetic testing.

Phenotypic evaluation identified affected relatives in 19 of GT-negative families (21%) and 42 GT-positive families (41%). Thus, despite a lower yield, more than one-fifth of families with negative GT had affected relatives who required structured follow-up. Predictive GT in GT-positive families identified 51% carriers, with a median of 2 relatives enrolled in specialized follow-up per proband (Figure 2). These data highlight the direct clinical impact of GT beyond the transplant recipient.

### Genetic susceptibility in cardiomyopathies attributed to “second-hit” causes

Forty-two patients were initially classified as having CM attributed to acquired or “second-hit” causes. Of these, 41 had a DCM phenotype and 1 had an ACM phenotype. A P/LP variant was identified in 36% of these patients overall, with subgroup yields of 33% (95% CI: 13%-62%) in postmyocarditis, 27% 95% CI: 8%-58% in chemotherapy-induced, 20% in alcohol-induced, 75% in tachycardia-induced, and 67% in peripartum CM (Table 2).

**Table 2:** Diagnostic Yield of Genetic Testing in Heart Transplant Recipients With Dilated Cardiomyopathy and a Preceding “Second-Hit” Etiology

Etiology	Genetic, n (%)	Genes
Myocarditis (n = 15)	5 (33)	*TTNx3, BAG3, DSP*
Chemotherapy-induced cardiomyopathy (n = 15)	4 (27)	*TTNx3, FLNC*
Alcohol-induced cardiomyopathy (n = 5)	1 (20)	*TTN*
Tachycardia-induced cardiomyopathy (n = 4)	3 (75)	*EMD, LAMP2, MYBPC3*
Peripartum cardiomyopathy (n = 3)	2 (67)	*FLNC, PKD2*
Total (n = 42)	15 (36)	*TTNx7, FLNCx2, BAG3, DSP, EMD, LAMP2, PKD2, MYBPC3*

Genetic findings in 42 HTx recipients with a clinical diagnosis of DCM attributed to a specific environmental or acquired insult (“second-hit”). P/LP variants were identified in 15 patients (overall diagnostic yield: 36%). Genes listed reflect the number of independent variant carriers; for example, “*TTN*x3” indicates 3 unrelated patients with a pathogenic *TTN* variant.

n = number of patients.

When restricted to the 41 patients with DCM phenotype, the diagnostic yield of GT in second-hit cases was 34% (14/41), which was lower than in the remaining DCM cohort (47%, 197/417; *P* = 0.10). This finding, although not statistically significant, supports a model in which genetic susceptibility contributes to progression toward ESHF even in the presence of identifiable environmental triggers.

### Genetic architecture of end-stage dilated cardiomyopathy differs from ambulatory cohorts

**Figure 3:**
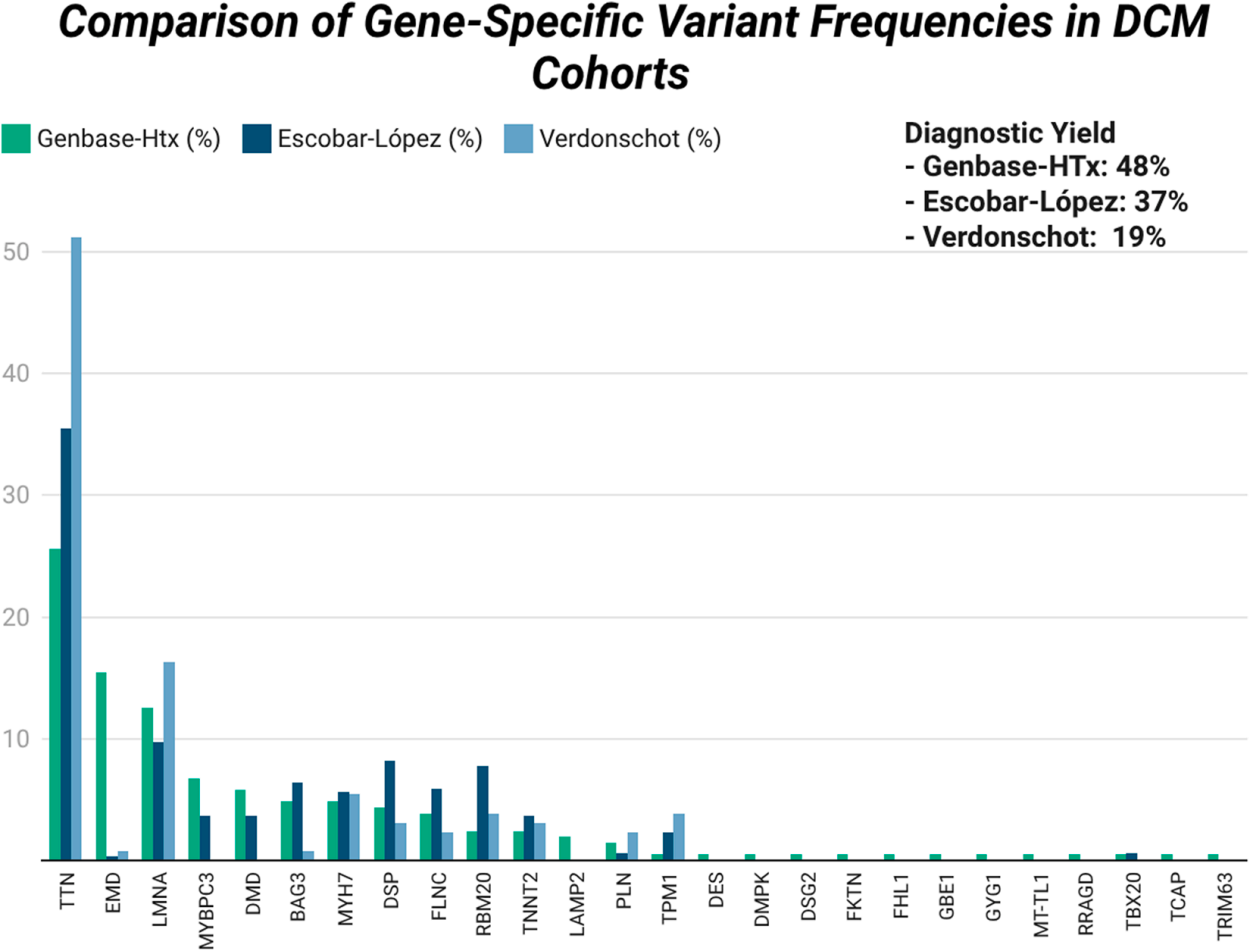
Age at Transplantation by Gene Group in Dilated Cardiomyopathy Bar graph comparing frequencies of pathogenic/likely pathogenic variants for major dilated cardiomyopathy-associated genes across 3 cohorts: the end-stage heart failure cohort (Genbase-HTx) and 2 general dilated cardiomyopathy cohorts (Escobar-López et al., Verdonschot et al). Variant frequencies are expressed as a percentage of genotype-positive patients within each cohort. DCM = dilated cardiomyopathy.

In DCM, *TTN* variants remained the most frequently identified gene but were significantly less prevalent than in ambulatory cohorts (25% vs 35%^14^ and 51%^15^ in two major references; both *P* < 0.01). Conversely, variants associated with high-risk arrhythmogenic phenotypes—including *LMNA, FLNC, DSP, PLN, TMEM43, and EMD*—were overrepresented. Notably, *EMD* variants were found in 15% of the DCM cohort (vs <1% in the ambulatory series; *P* < 0.01), reflecting a specific founder effect in the Spanish population^8^^,^^16^ (Figure 3, Supplemental Tables 8 and 9).

**Figure 4:**
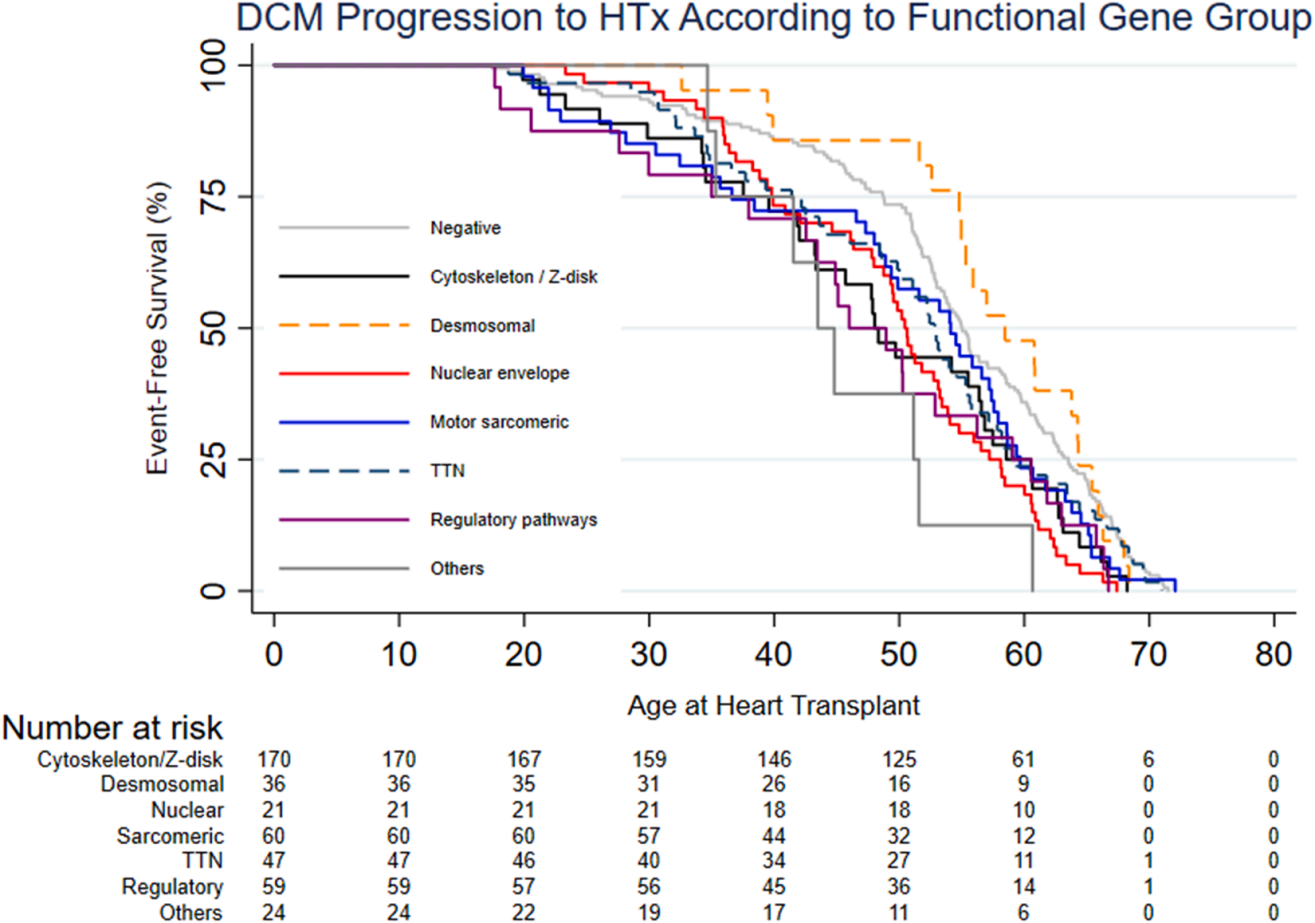
Gene Variant Frequencies Across Dilated Cardiomyopathy Cohorts Kaplan-Meier curves showing transplant-free survival according to functional gene groups in patients with dilated cardiomyopathy. Age at heart transplantation was used as the time scale. Numbers at risk are shown below the plot. DCM = dilated cardiomyopathy; other abbreviation as in Figure 3.

**Central Illustration:**
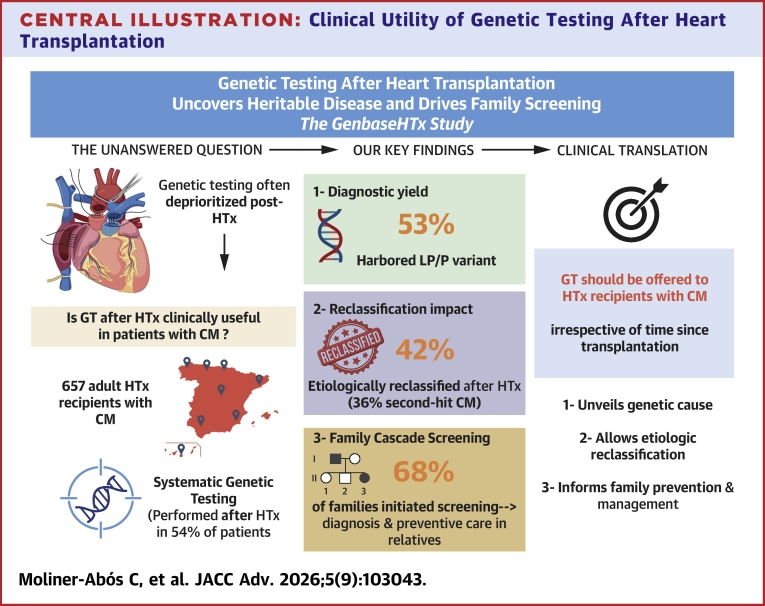
Clinical Utility of Genetic Testing After Heart Transplantation In 657 heart transplant recipients with cardiomyopathy, systematic genetic testing identified a pathogenic variant in 53%, led to etiologic reclassification in 42% of those tested post-transplant, and activated family cascade screening in 69% of families. These findings support integrating genetic testing into long-term care of all transplant recipients with cardiomyopathy. Created using BioRender.com. CM = cardiomyopathy; HTx = heart transplantation; other abbreviation as in Figure 2.

Genetic background was a major determinant of the timing of progression to ESHF. After adjusting for between-center heterogeneity and temporal cohort, the presence of a positive GT remained significantly associated with accelerated progression to heart transplantation (HR = 1.30; 95% CI: 1.06-1.58; *P* = 0.010).

After adjusting for between-center heterogeneity and temporal cohort and stratified by biological functional groups, nuclear envelope genes (HR: 1.78; 95% CI: 1.31-2.43), regulatory pathway genes (including *BAG3, RBM20*, and *PLN*; HR: 1.59; 95% CI: 1.01-2.52), cytoskeleton/Z-disk genes (HR: 1.53; 95% CI: 1.05-2.24) and other rare variants (HR: 3.08; 95% CI: 1.54-6.18) remained significantly associated with accelerated progression to transplantation (Figure 4, Supplemental Figure 3). In contrast, carriers of motor sarcomeric or *TTN* variants exhibited a clinical trajectory that did not differ significantly from genotype-negative individuals.

Patients harboring arrhythmogenic variants (*LMNA, FLNC, DSP, PLN, TMEM43, EMD*) reached HTx at a significantly younger age than genotype-negative controls (HR: 1.50; 95% CI: 1.15-1.05; *P* = 0.03; Supplemental Figure 4). These findings identify specific genetic pathways that dictate a high “disease velocity” toward terminal heart failure.

While this analysis focused on DCM, phenotype-specific genetic patterns were also observed in HCM, ACM, and RCM. In HCM, *MYBPC3* and *MYH7* accounted for ∼60% of positive cases, similar to ambulatory cohorts. In end-stage ACM, we observed a higher prevalence of *DSG2*, *LMNA*, and *TMEM43*, and a lower prevalence of *PKP2* and *DSP*, compared with published ambulatory data. In RCM, transthyretin predominated (48% of positive cases), whereas ambulatory RCM is typically dominated by *MYH7* (detailed in Supplemental Tables 10-12 and Supplemental Figures 5-7).

Together, these findings not only characterize end-stage disease but also identify specific genetic pathways associated with accelerated progression, which could inform future risk stratification and targeted therapeutic strategies in dilated CM.

### Additional genetic findings

Multiple P/LP variants were identified in 5% of genetically positive patients, including digenic and compound heterozygous genotypes (Supplemental Table 13). CNVs were detected in 3% of patients undergoing NGS-based GT. Age at HTx was similar between patients with multiple variants and those with a single variant (median 50.4 [IQR: 39.9-58.5] vs 52.7 [IQR: 42.0-59.8] years, *P* = 0.73), as was the proportion of patients listed as urgent (25% vs 32%, *P* = 0.61). Notably, the 5 patients with homozygous genotypes reached HTx at a markedly younger age than both single-variant carriers (median 31.5 [IQR: 17.4-44.8] vs 52.7 years, *P* < 0.01) and patients with multiple nonhomozygous variants (median 31.5 vs 50.4 years, *P* = 0.02), suggesting a gene-dosage effect wherein biallelic variants confer particularly high disease severity.

## Discussion

In this nationwide multicenter study of adult HTx recipients with CM, we demonstrate that GT remains clinically actionable even when performed years after transplantation. Genetic evaluation led to etiologic reclassification in nearly one-third of patients, activated family cascade screening in more than half of families, and uncovered a genetic architecture of ESHF that differs from that described in ambulatory CM cohorts. Together, these findings challenge the prevailing assumption that etiologic investigation loses relevance after HTx and support the systematic integration of GT into the long-term care of HTx recipients (Central Illustration).

### Genetic testing after heart transplantation is not “too late”

Once a pathogenic variant is identified in a CM patient, genetic cascade screening of relatives is a cost-effective cornerstone of care.^2^^,^^17^^,^^18^ It accurately identifies at-risk individuals who require lifelong surveillance while safely discharging noncarriers from further evaluation.

Current guidelines^2^^,^^3^ recommend GT for patients with CM, primarily emphasizing ambulatory populations and early disease stages. In clinical practice, however, etiologic investigation is frequently deprioritized once HTx has occurred, under the assumption that genetic information no longer influences patient management. Our data directly challenge this paradigm. Importantly, we observed a significant shift in clinical practice over the study period, with pretransplant GT increasing from 12% in 2010 to 2014 to 68% in 2020 to 2023 (*P* for trend <0.001), reflecting growing recognition of the value of genetic information in end-stage disease. Despite this positive trend, more than half of patients underwent testing after transplantation—a median of 4.3 years post-HTx—and this “delayed” testing still led to etiologic reclassification in 42% of cases. These findings establish GT as a clinically relevant and actionable tool even in advanced disease stages and long after transplantation, with direct implications for preventive cardiology and inherited disease management.

### Genetic susceptibility underlies a substantial proportion of “second-hit” cardiomyopathies

A significant proportion of CMs progressing to ESHF are attributed to acquired or environmental factors such as chemotherapy exposure, myocarditis, alcohol abuse, or tachycardia-mediated dysfunction. These etiologies are often conceptualized as nongenetic, leading to the omission of GT from diagnostic workflows.

In our cohort, more than one-third of CMs initially attributed to such “second-hit” causes harbored a pathogenic genetic variant. This yield is substantial and aligns with the growing recognition of a genetic predisposition in seemingly “secondary” CMs.^19^, ^20^, ^21^, ^22^

Notably, our findings are consistent with recent evidence regarding inflammatory phenotypes; a 2024 meta-analysis^22^ reported a 21.9% prevalence of P/LP variants in adults with complicated myocarditis. By demonstrating an even higher yield in our heart transplant population, we confirm that severe clinical trajectories—often labeled as purely inflammatory or toxic—frequently represent the clinical expression of an underlying genetic vulnerability.

Furthermore, the genetic architecture observed in our postmyocarditis subgroup (n = 15; 33% yield) provides mechanistic insight into disease progression toward ESHF. While desmosomal variants are frequently linked to uncomplicated or arrhythmogenic myocarditis, complicated cases presenting with severe heart failure have been associated with a predominance of sarcomeric genes. Consistent with this, 80% of our genotype-positive myocarditis cases harbored variants in structural or sarcomeric genes (*TTN* and *BAG3*), with only one case involving a desmosomal gene (*DSP*).

This finding supports a model in which genetic susceptibility interacts with environmental stressors to accelerate disease progression toward ESHF. From a clinical standpoint, these findings highlight the importance of not overlooking GT, even in the presence of an identifiable cause that could account for CM. A structural genetic “first-hit” may be a prerequisite for the aggressive remodeling and rapid functional decline observed in patients who ultimately require HTx following an inflammatory or toxic insult, particularly when disease severity is disproportionate or progression is rapid. We therefore propose that GT should be considered in any patient presenting with ESHF and a history of an apparent environmental trigger.

### Genetic testing in families with negative results: clinical surveillance remains essential

In 64 patients, GT did not identify a monogenic cause in families with clinically suspected familial CM. This finding requires careful interpretation. First, initial classification as “familial” was often based on information from the national transplant registry without independent verification of family history—a common limitation in real-world practice. Second, even when family history was reported, confirming true CM or SCD in relatives is frequently hindered by lack of medical records or autopsy data.

Conversely, FS also plays a critical role in refining variant interpretation. In several families from our cohort, segregation analysis through family evaluation provided evidence that enabled reclassification of variants initially classified as variant of uncertain significance to LP/P. This bidirectional value of FS—identifying at-risk relatives while simultaneously clarifying the clinical significance of identified variants—highlights its indispensability in the management of heritable CM.

Current genomic knowledge is incomplete, with potentially undiscovered genes and noncoding variants yet to be characterized. Moreover, our analysis focused on monogenic causes, which does not exclude the contribution of polygenic factors^23^ or intermediate-effect variants^24^ that may cluster in families. Therefore, families with clear aggregation but negative comprehensive GT should continue to undergo periodic clinical surveillance and be considered for research-based approaches to identify novel genetic mechanisms. This distinction is particularly relevant for clinical practice: while predictive GT is only possible in families with an identified variant, clinical screening remains essential in all families with suspected heritable disease, regardless of genetic results.

### The genetic architecture of end-stage dilated cardiomyopathy reflects accelerated disease pathways and informs a new natural history

Beyond diagnostic yield, our study provides unique insight into the biology of CM progression to its most extreme stage. In contrast to ambulatory DCM cohorts,^14^^,^^15^ transplanted patients exhibited a lower prevalence of *TTN* variants and a significant enrichment of genes associated with arrhythmogenic and high-risk phenotypes (*LMNA*, *FLNC*, desmosomal genes, *RBM20, PLN*). This observation aligns with and extends recent evidence from nontransplanted cohorts, where patients with these same high-risk arrhythmic genotypes experience a markedly increased rate of advanced heart failure events, including transplantation.^25^

A pivotal implication of our findings, viewed in this context, is the illustration of a shifted clinical trajectory in the modern era. With the widespread use of primary prevention implantable cardioverter-defibrillators in patients with high-risk genetic substrates, SCD is effectively mitigated. Consequently, the inherent progressive myocardial dysfunction of these specific genotypes becomes the dominant and unmasked clinical challenge, culminating in ESHF. Our cohort provides the phenotypic confirmation of this pathway at its ultimate endpoint. This underscores that genetic diagnosis in DCM is not solely a tool for arrhythmic risk stratification but also identifies a subset of patients destined for accelerated heart failure progression, who may benefit from earlier and more aggressive heart failure management.

Importantly, functional gene group analysis further refined this concept. Variants affecting nuclear envelope proteins and regulatory pathways (e.g., RNA processing, calcium handling) were associated with younger age at HTx, while sarcomeric and *TTN*-related variants were not. These findings support a model of genotype-specific disease velocity and reinforce the need for a dual-track management strategy in genetic DCM: one for arrhythmia prevention and another for proactive surveillance and timely intervention for heart failure progression.

Our observations are in line with, and extend, prior studies of GT in HTx recipients (see Supplemental Table 14 for a summary of published series),^5^, ^6^, ^7^, ^8^ by providing a mechanistic and clinical framework for the genetic landscape of end-stage disease. The higher yield in our cohort (53%) compared with previous reports (36% to 44%) is explained by the use of larger NGS panels, systematic CNV assessment, family cosegregation analysis, a recurrent *EMD* founder effect, and methodological differences detailed in Supplemental Table 13.

## Clinical implications

Our findings support a shift in the management of advanced heart failure. GT is essential for all HTx recipients with CM, regardless of time since transplantation, to enable precise reclassification and to activate lifesaving FS. Patients with presumed “second-hit” etiologies should not be excluded from genetic evaluation, as underlying susceptibility is a major driver of progression to end-stage disease.

The genetic architecture of ESHF is dominated by high-risk arrhythmogenic genotypes (e.g., *LMNA*, *FLNC*). This demands a proactive management strategy: carriers of these variants should be referred early to specialized heart failure units for close monitoring to detect the transition to overt systolic dysfunction. This allows for the prompt initiation and aggressive titration of neurohormonal therapy, even in the presence of mild ventricular impairment, to potentially delay progression toward transplantation.

Ultimately, our cohort defines the genetic landscape of terminal myocardial failure. This provides a rationale for developing genotype-specific therapies^26^ aimed at preventing progression to transplantation, and argues that future clinical trials for these genotypes must prioritize heart failure outcomes alongside arrhythmic endpoints.

### Study limitations

This study has several limitations. Its retrospective design introduces the possibility of selection bias, and GT methodologies evolved over the study period. Moreover, the final cohort represents 56% of all CM-related transplantations at participating centers. A comparison of baseline characteristics between included and excluded patients (Supplemental Table 3) showed that included patients were significantly younger and more likely to belong to recent temporal cohorts, reflecting increased GT adoption over time, while no significant differences were observed for sex or primary etiology. However, we cannot exclude the possibility of residual selection bias, as GT may have been preferentially ordered in patients with higher pretest probability of genetic disease—a factor not fully captured by available baseline variables. FS data were not available for all patients, potentially underestimating the downstream impact of GT. Additionally, comparisons with ambulatory cohorts rely on published data rather than direct contemporaneous controls. The high prevalence of *EMD* variants (15%) reflects a known founder effect in the Spanish population and limits the generalizability of this specific finding to other geographic regions. However, the remaining genetic architecture—including the relative depletion of *TTN* and enrichment of arrhythmogenic genes—likely reflects biological determinants of progression to end-stage disease applicable across populations. Furthermore, our definition of etiologic reclassification includes both true diagnostic changes and etiologic specification, which may overestimate the clinical impact from a strict phenotypic perspective. However, even etiologic specification has clinical relevance for family screening and prognostic refinement. Finally, associations between genetic subgroups and age at HTx should be interpreted as hypothesis-generating.

## Conclusions

GT performed after HTx identifies clinically actionable heritable CM in a substantial proportion of patients, enables etiologic reclassification, and drives family cascade screening. The genetic architecture of CM progressing to ESHF differs from that of ambulatory disease, with specific functional gene groups associated with accelerated progression. These findings support the routine use of GT in HTx recipients with CM, irrespective of the presence of environmental triggers or the timing of testing relative to transplantation.

## Funding support and author disclosures

Dr Moliner-Abós received a Río Hortega grant (CM21/00245) from the 10.13039/501100004587Instituto de Salud Carlos III, Madrid, Spain. This work was supported by the Spanish Ministerio de Economia y Competitividad, Madrid, Spain; 10.13039/501100004587Instituto de Salud Carlos III, Madrid, Spain; 10.13039/501100012513Centro de Investigación Biomédica en Red Enfermedades Cardiovaculares, CIBERCV (CB16/11/00276), Madrid, Spain; and the 10.13039/501100008530Fondo Europeo de Desarrollo Regional (FEDER). The authors have reported that they have no relationships relevant to the contents of this paper to disclose.
