# Supplementary material for: Genetic Testing After Heart Transplantation Uncovers Heritable Disease and Drives Family Screening
Source: JACC Adv. 2026 Jul 31;5(9):103043. doi: 10.1016/j.jacadv.2026.103043 (PMC13452982; doi:10.1016/j.jacadv.2026.103043)
Supplement: Supplementary file 1 [file mmc1.pdf]

Genetic Testing After Heart Transplantation Uncovers Heritable Disease and Drives Family Screening

SUPPLEMENTAL MATERIAL

Figures

Figure S1. Study Cohort Derivation Flowchart

Figure S2. Trends in Pre-Transplant Genetic Testing

Figure S3. Association Between Functional Gene Groups and Age at Heart Transplantation in Patients With Dilated Cardiomyopathy

Figure S4. Impact of Arrhythmogenic Genetic Profile on Age at Heart Transplantation in Patients With Dilated Cardiomyopathy

Figure S5. Comparison of Gene Specific Variant Frequencies Across HCM Cohorts

Figure S6. Comparison of Gene Specific Variant Frequencies Across ACM Cohorts

Figure S7. Comparison of Gene Specific Variant Frequencies Across RCM cohorts.

Tables

Table S1. List of all variants with ACMG classification

Table S2. Missing data patterns in the survival analysis sample

Table S3. Baseline characteristics of included vs. excluded participants

Table S4. Baseline characteristics by genetic testing results

Table S5. Diagnostic Yield by Phenotype and Cohort Type

Table S6. Cardiac and Skeletal Muscle Phenotypes

Table S7. Reclassification of Initial Clinical Etiology

Table S8. Distribution of Pathogenic Variants and Genetic Testing Positivity in Dilated Cardiomyopathy Cohorts

Table S9. Comparison of Pathogenic Gene Variant Distribution Across Dilated Cardiomyopathy Cohorts: General vs. End-Stage Heart Failure Cohorts

Table S10. Distribution and comparison of gene variants in ambulatory versus end-stage HCM cohorts

Table S11. Distribution and comparison of gene variants in ambulatory versus end-stage ACM cohorts

Table S12. Distribution and comparison of gene variants in ambulatory versus end-stage RCM cohorts

Table S13. Heart Transplant Recipients Carrying Multiple Pathogenic or Likely Pathogenic Variants

Table S14. Summary of Prior Studies Assessing the Diagnostic Yield of Genetic Testing in Heart Transplant Recipients With Cardiomyopathy

Figure S1. Study Cohort Derivation Flowchart

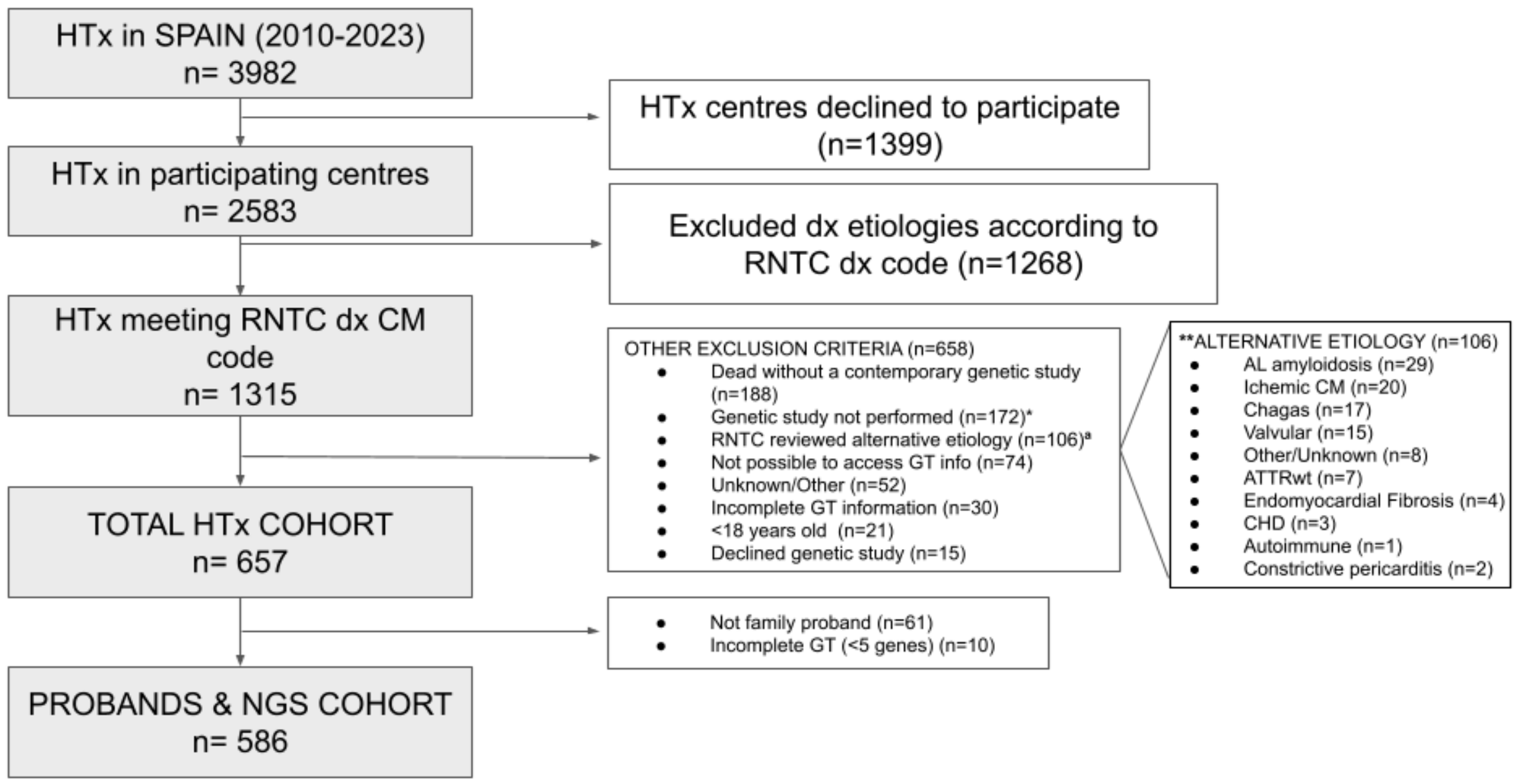

From all adult heart transplant recipients (HTx) recorded in the Spanish National Heart Transplant Registry (RNTC; 2010–2023), the final analytic cohort comprised 657 cardiomyopathy (CM) patients who underwent comprehensive genetic testing (GT). [a]Includes 14 patients with myocarditis and 11 patients with anthracycline-induced CM who were not tested due to local practices. *N indicates number of patients. NGS, next-generation sequencing; ATTRwt, wild-type cardiac amyloidosis.

Figure S2. Trends in Pre-Transplant Genetic Testing

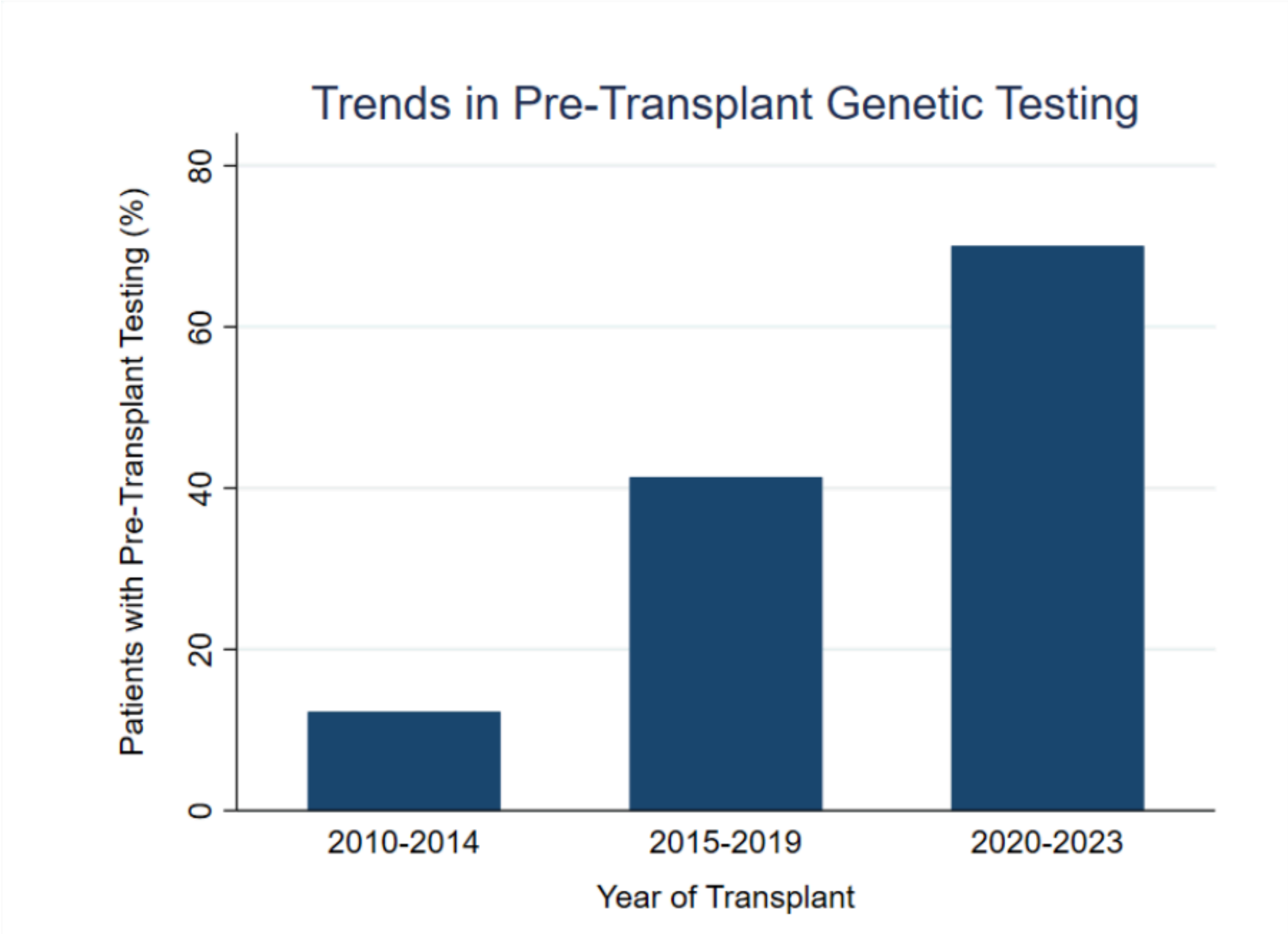

Bar graph showing the percentage of patients who underwent genetic testing before heart transplantation across three periods: 2010-2014, 2015-2019, and 2020-2023. Data represent the 474 patients (72% of the total cohort) for whom timing of genetic testing was available. The proportion of pre-transplant testing increased from 12% (13/106) in 2010-2014 to 41% (79/191) in 2015-2019, reaching 68% (124/177) in 2020-2023 (p for trend <0.001).

Figure S3. Association Between Functional Gene Groups and Age at Heart Transplantation in Patients With Dilated Cardiomyopathy

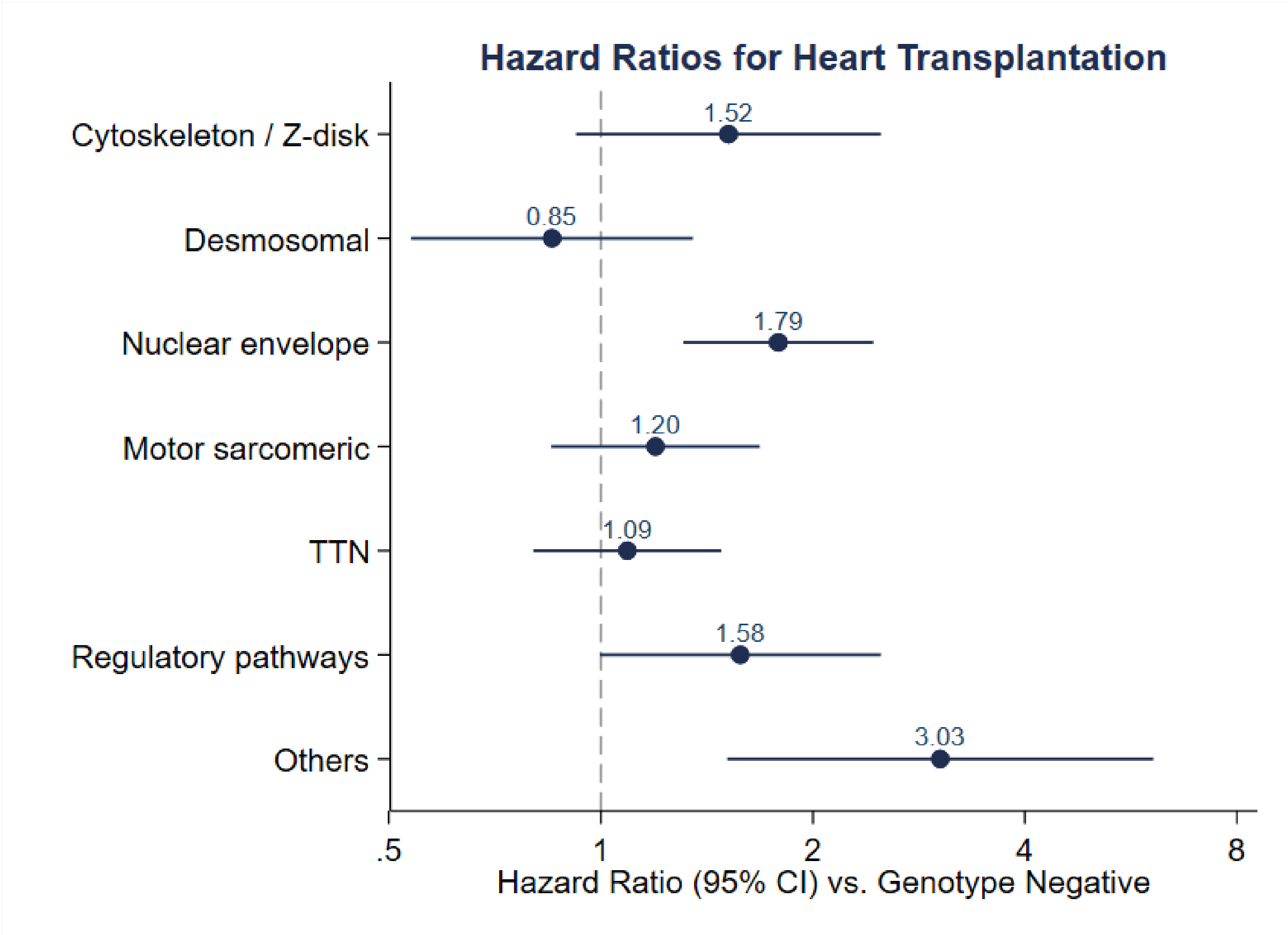

This forest plot illustrates the risk of progression to heart transplantation in DCM patients stratified by genetic functional groups. *"Cytoskeleton/Z-disk (FLNC, JPH2); Desmosomal (DSC2, DSG2, DSP, PKP2); Nuclear envelope (EMD, LMNA, TMEM43); Motor sarcomeric (ACTC1, MYBPC3, MYH7, TNNC1, TNNI3, TNNT2, TPM1); TTN; Regulatory pathways (BAG3, PLN, RBM20). DMD was excluded due to distinct clinical behavior."* Abbreviations: CI = confidence interval; DCM = dilated cardiomyopathy; HR = hazard ratio; HTx = heart transplantation.

Figure S4. Impact of Arrhythmogenic Genetic Profile on Age at Heart Transplantation in Patients With Dilated Cardiomyopathy

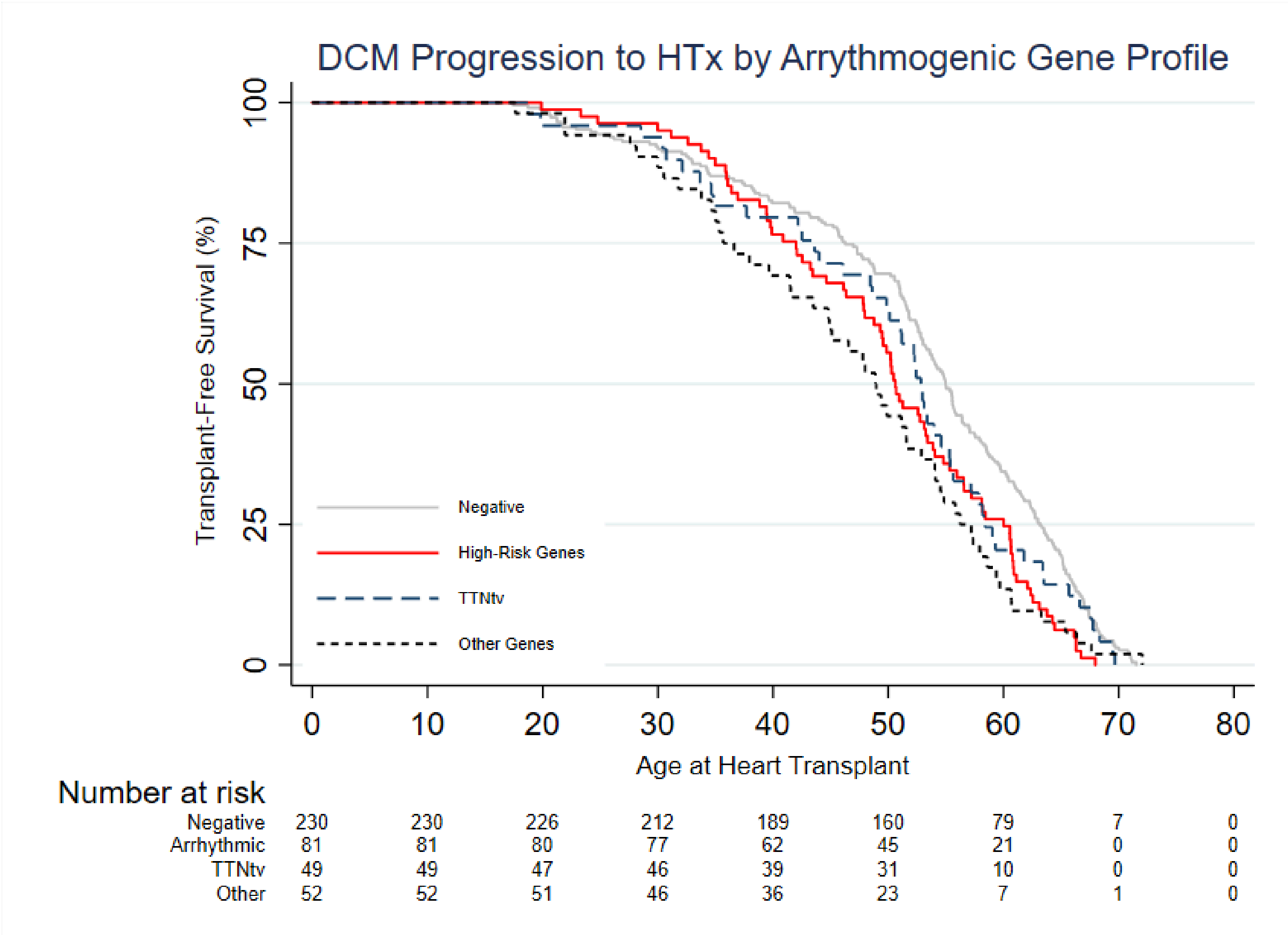

Kaplan–Meier curves showing transplant-free survival according to arrhythmogenic genetic risk profiles in patients with dilated cardiomyopathy (DCM). Patients were stratified into four groups: high-risk arrhythmogenic (including *LMNA, FLNC, EMD, RBM20,* and desmosomal genes), *TTNtv* carriers, other genetic etiologies, and genotype-negative patients. Age at heart transplantation was used as the time scale to reflect disease progression and the timing of end-stage heart failure. Log-rank testing demonstrated significantly earlier transplantation in the high-risk arrhythmogenic group compared to *TTNtv* and genotype-negative individuals ($p < 0.001$). Numbers at risk are shown below the plot.

Figure S5. Comparison of Gene Specific Variant Frequencies Across HCM Cohorts

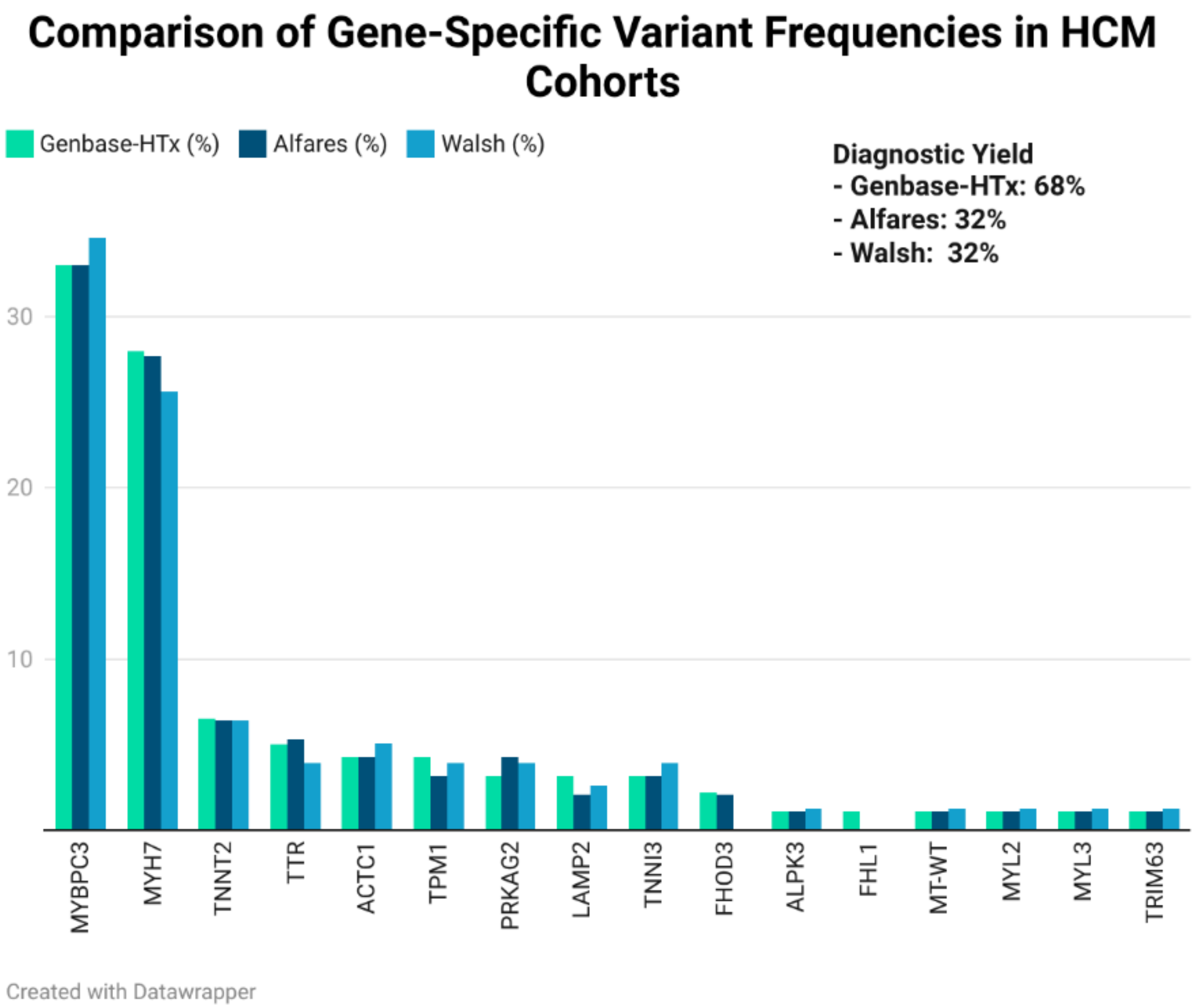

Bar graph comparing the frequency of pathogenic and likely pathogenic variants for key cardiomyopathy-associated genes between the Genbase-Htx, Alfares et al. and Walsh et al. cohorts of patients with hypertrophic cardiomyopathy (HCM). Variant frequencies are expressed as a percentage of genotype-positive patients within each cohort.

Figure S6. Comparison of Gene Specific Variant Frequencies Across ACM cohorts.

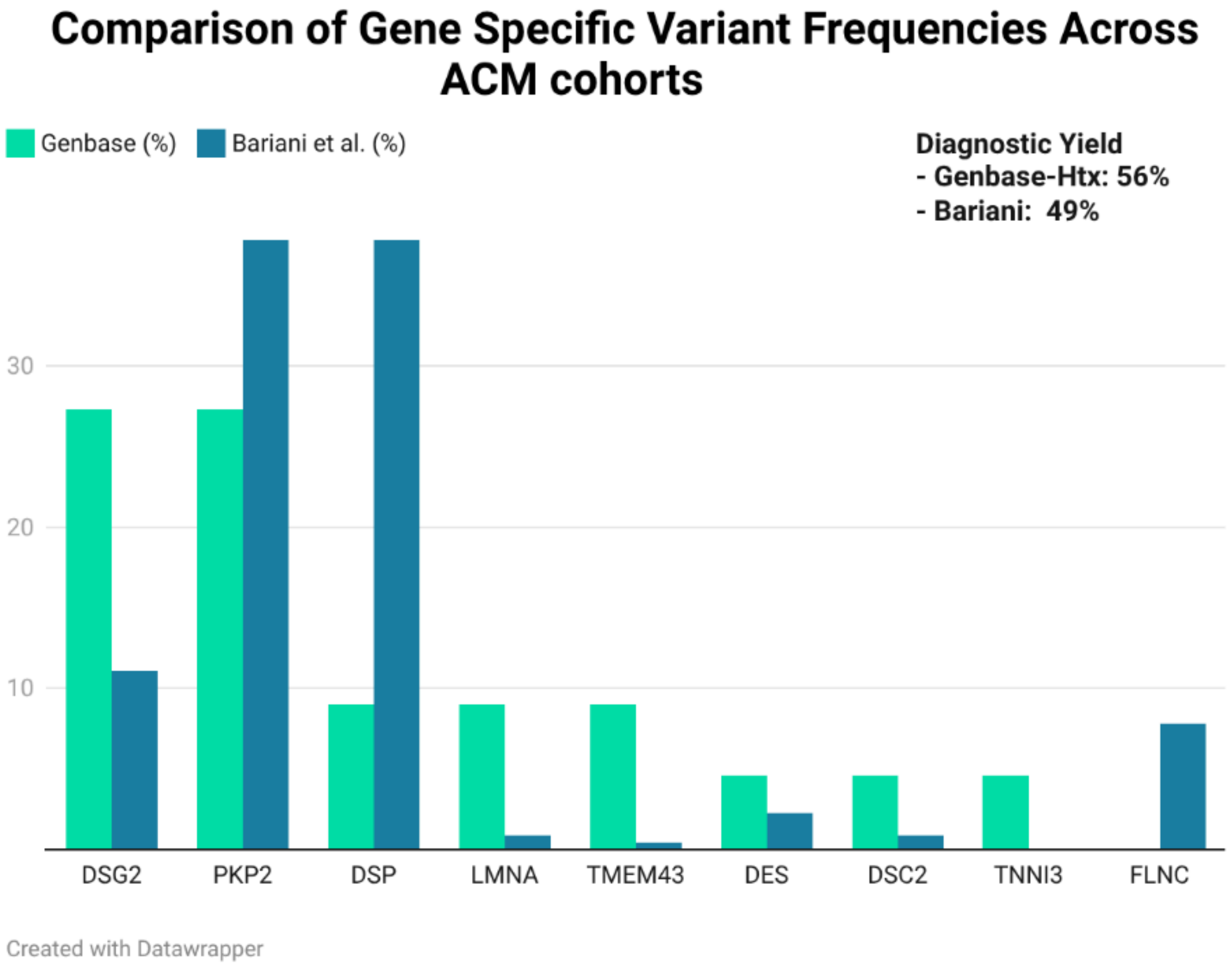

Bar graph comparing the frequency of pathogenic and likely pathogenic variants for key cardiomyopathy-associated genes between the Genbase-Htx and Bariani et al. cohorts of patients with arrhythmogenic cardiomyopathy (ACM). Variant frequencies are expressed as a percentage of genotype-positive patients within each cohort.

Figure S7. Comparison of Gene Specific Variant Frequencies Across RCM cohorts.

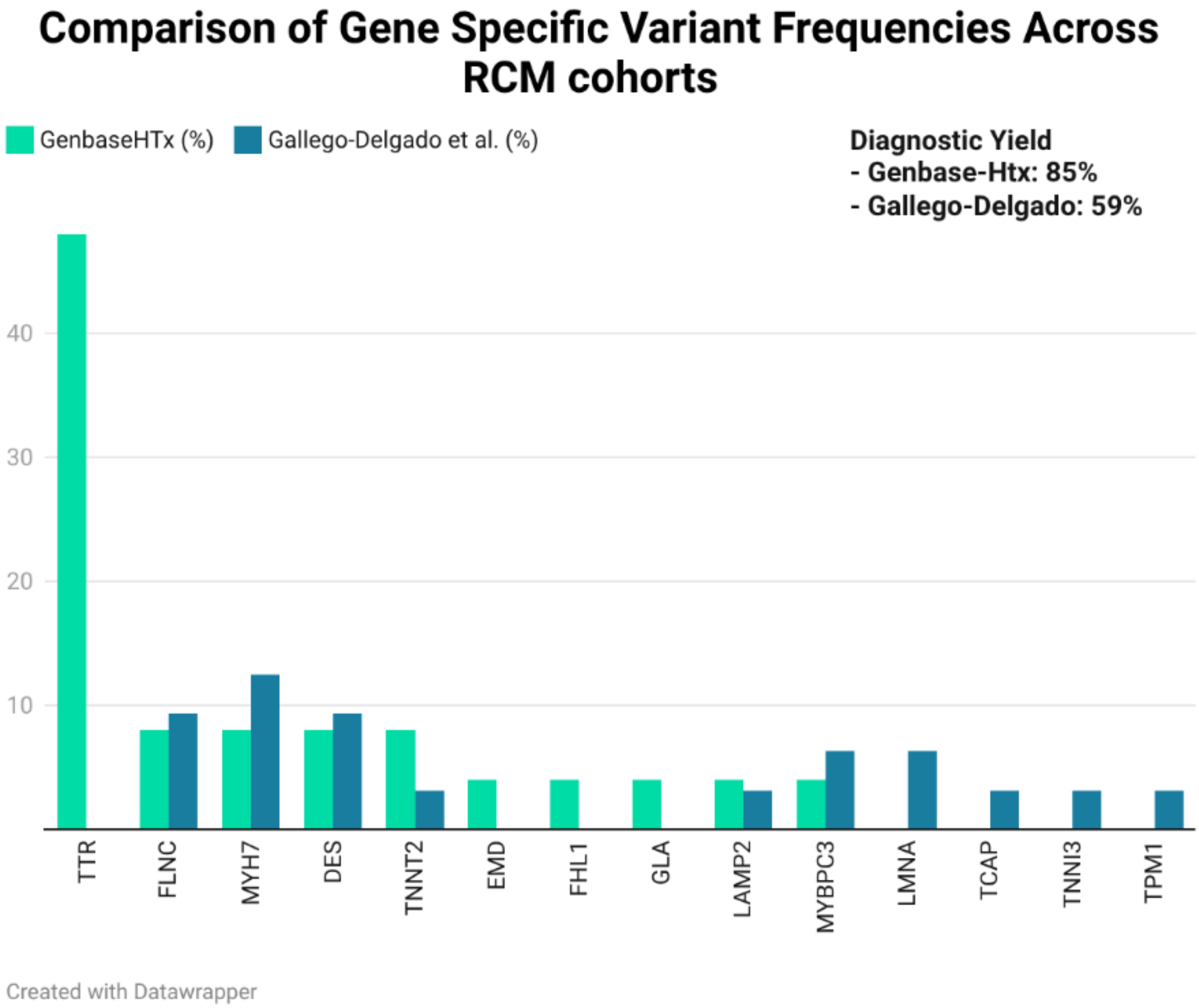

Bar graph comparing the frequency of pathogenic and likely pathogenic variants for key cardiomyopathy-associated genes between the Genbase-Htx and Gallego-Delgado et al. cohorts of patients with restrictive cardiomyopathy (RCM). Variant frequencies are expressed as a percentage of genotype-positive patients within each cohort.

Table S1. List of all variants with ACMG classification and criteria

Variant type	Gene	Transcript	cDNA change	Protein change	ACMG class	ACMG criteria
Truncating - Frameshift	DSP	NM_004415.4	c.8208dupT	p.Val2737Cysfs*2	LP	PM2, PVS1_mod, PS4_supp, PP1
Missense	FKTN	NM_001079802.1	c.1270G>A	p.Gly424Ser	LP	PM2_supp, PP3, PM3, PP1_mod
Missense	FHL1	NM_001159700.1:c.763T>C	NC_000023.10:g.135292104T>C	p.Cys255Arg	LP	PM2_Supp, PS4_Supp, PM5, PP4 y PP3.
Missense	FLNC	NM_001458.4	c.4874C>T	p.Pro1625Leu	LP	PM2_supp, PS4_mod, PP3, PP1
Truncating - Frameshift	DSP	NM_004415.3	c.6689delCinsTGGTATATCCA	p.Pro2230Leufs*9	LP	PM2, PVS1_strong
CNV (deletion)	LAMP2	NM_002294.2	c.-33399_1093+2541delins174	p.0?	LP	1A, 2A

Splice-site (canonical ±1/±2)	LAMP2	NM_002294.2	c.864+1G>T		LP	PM2_supp, PVS1_mod, PS1
Missense	TPM1	NM_000366.6	c.109A>G	p.Lys37Glu	LP	PM2, PS4_supp, PP2, PP1_mod
Truncating - Frameshift	TTN	NM_003319.4	c.40460_40461insT	p.Leu13487Phefs*6	LP	PVS1, PM2_supp
Truncating - Frameshift	LAMP2	NM_002294.3	c.705_708del	p.Thr236Trpfs*5	LP	PM2_supp, PVS1
Truncating - Frameshift	DMD	NM_004006.3	c.10362_10366dup	p.Asn3456Ilefs*2	LP	PM2_supp, PVS1
Missense	MYH7	NM_000257.4	c.1618T>C	p.Phe540Leu	LP	PM2, PP3, PM1, PS4_supp
Missense	TTR	NM_000371.4	c.193G>A	p.Ala65Thr	LP	PM2, PP3, PS4_mod, PM5
Truncating - Frameshift	TTN	NM_003319.4	c.49954_49955delCT	p.Leu16652Glufs*6	LP	PM2_supp, PVS1

Missense	TTR	NM_000371.4	c.193G>A	p.Ala65Thr	LP	PM2, PP3, PS4_mod, PM5
Intronic affecting splicing	DSG2	NM_001943.5	c.523+4A>G		LP	PM2_supporting, PS4, PS1_moderate, PP3
Truncating - Frameshift	TTN	NM_003319.4	c.39478_39479del	p.Asp22225Hisfs*11	LP	PM2_supp, PVS1
Truncating - Nonsense	DSG2	NM_001943.5	c.1494del	p.Leu499*	LP	PM2_supp, PVS1
Missense	LMNA	NM_170707.4	c.370G>A	p.Glu124Lys	LP	PM2_supporting, PS4, PP1_moderate y PP3
Missense	GBE1	NM_000158.3	c.759A>G	p.Thr254Ala	LP	PM2_supp, PM3_strong, PP3, PS3_supp
Missense	TTR	NM_000371.4	c.289T>A	p.Ser97Thr	LP	PM2_supp, PS4_mod, PM5, PP4

Truncating - Nonsense	TTN	NM_003319.4	c.30523C>T	p.R10175*	LP	PM2_supp, PVS1
Missense	TTR	NM_000371.4	c.112G>A	p.Asp38Asn	LP	PM2, PP3, PS4_mod, PM5
Missense	TNNI3	NM_000363.4	c.569A>T	p.Asp190Val	LP	PM2, PP3, PM1, PS4_supp
Missense	RRAGD	NM_021244.5	c.227C>T	p.Ser76Leu	LP	PM2, PP3, PP2, PS4_supp, PM6
Missense	MYH7	NM_000257.3	c.709C>T	p.Arg237Trp	LP	PM2_supp, PS4_mod, PM1, PS3_supp
Truncating - Frameshift	TTN	NM_003319.4	c.16492_16493del	p.Thr5498Leufs*4	LP	PM2_supp, PVS1
Truncating - Frameshift	TTN	NM_003319.4	c.25872_25875dup	p.Arg8626Serfs*2	LP	PM2_supp, PVS1
Truncating - Frameshift	FLNC	NP_001449.3	c.1414delT	p.Cys472Valfs*20	LP	PM2_supp, PVS1

Missense	LMNA	NM_170707.4	c.1621C>T	p.Arg541Cys	LP	PM2_supp, PS4_smod, PP3, PM6, PP1
Missense	TPM1	NM_001018006.1	c.337C>G	p.Leu113Val	LP	PM2_supp, PS4_mod, PP2, PP3, PP1
Truncating - Nonsense	TTN	NM_003319	c.21304C>T	p.Arg7102*	LP	PM2_supp, PVS1
Truncating - Frameshift	TTN	NM_0003319	c.62569_62570del	p.Lys20857Valfs*7	LP	PM2_supp, PVS1
Truncating - Frameshift	LMNA	NM_170707	c.619del	p.Gln207Argfs*273	LP	PM2_supp, PVS1
Missense	TPM1	NM_000366	c.841A>G	p.Met281Val	LP	PS4, PP2, PM5
Truncating - Frameshift	DSP	NM_004415.4	c.423del	p.Arg141Serfs*55	LP	PM2_supp, PVS1

Missense	PRKAG2	NM_016203.4	c.1004T>C	p.Met335Thr	LP	PM2_Supporting, PS4_Moderate, PM1, and PP3
Truncating - Frameshift	LMNA	NM_170707	c.619del	p.Gln207Argfs*273	LP	PM2_supp, PVS1
Truncating - Frameshift	LMNA	NM_170707	c.619del	p.Gln207Argfs*273	LP	PM2_supp, PVS1
Truncating - Frameshift	MYBPC3	NM_000256.3	c.2190delC	p.Thr730fs*24	LP	PM2_supp, PVS1
Truncating - Frameshift	LAMP2	NM_002294.2	c.1054delG	p.Val352Phefs*6	LP	PM2_supp, PVS1_strong, PS4_supp
Truncating - Frameshift	LAMP2	NM_002294.2	c.714del	p.Leu239Cysfs*3	LP	PM2_supp, PVS1
Truncating - Frameshift	DSP	004425.4	c.5316delT	p.Leu1773fs*8	LP	PM2_supp, PS4, PVS1_strong

Missense	MYH7	NM_000257.3	c1498G>A	p.Glu500Lys	LP	PM2, PP3, PS4_sup, PM1
Delins	DSP	NM_004415.3	c.1909_1910insTCATGTAAATCACCATGATTTTACAGTGAC	p.Thr637_His2871delinsIleMet *	LP	PVS1, PM2_supp
Missense	TTR	NM_000371.3	c.128G>A	p.Ser43Asn	LP	PM2_Supporting, PS4 y PP1_Strong
Missense	TTR	NM_000371.3	c.128G>A	p.Ser43Asn	LP	PM2_Supporting, PS4 y PP1_Strong
Truncating - Frameshift	DSP	NM_004415.3	c.1909_1910insTCATGTAAATCACCATGATTTTACAGTGAC	p.Thr637_His2871delinsIleMet*	LP	PM2_supp, PVS1

Truncating - Nonsense	BAG3	NM_004281.3	c.358C>T	p.Gln120*	LP	PVS1/PM2_supp
Splice-site (canonical ±1/±2)	PKP2	NM_004572.3	c.2578-2A>C		LP	PM2_supp, PVS1_mod, PS4_mod
Splice-site (non-canonical intronic)	MYBPC3	NM_000256.3	c.2737+4_2737+7delAGTG		LP	PM2_supp, PP3, PS1 y PS4_supp
Truncating - Frameshift	MYBPC3	NM_000256.3	c.3437_3438insTTGG	p.Phe1147Trpfs*3	LP	PM2_supporting, PVS1 y PP1
Missense	TCAP	NM_003673.3	c.473G>A	p.Arg158His	LP	PM2_supp, PS4_mod, PP3 y PM5.
Missense	MYH7	NM_000257.2	c.1452G>C	p.Lys484Asn	LP	PM2_supporting,PP1_moderate,PM1,PP3_supporting
Truncating - Nonsense	TTN	NM_003319.4	c.54853A>T	p.Lys18285*	LP	PVS1, PM2_supp

Truncating - Nonsense	LMNA	NM_170707.3	c.1377_1378insT	p.Glu460*	LP	PM2_supp, PVS1
Missense	MYH7	NM_000257.4	c.1030G>A	p.Glu344Lys	LP	PM2_supp, PS4_supp, PP3_mod y PM1
Missense	GYG1	NM_004130.4	c.304G>C	p.Asp102His	LP	PM3_strong, PS3_moderate
Truncating - Nonsense	DMD	NM_004006.3	c.4069G>T	p.Glu1357*	LP	PM2_supporting y PVS1
Truncating - Frameshift	TTN	NM_003319.4	c.56232_56233insTTGTCGAAAAACGATAAACTACA	Arg18745Leufs*69	LP	PM2_supporting y PVS1
Truncating - Frameshift	TTN	NM_003319.4	c.66240delA	p.Asp22081Metfs*31	LP	PM2_supporting y PVS1
In-frame deletion	MYH7	NM_000257.4	c.2539_2541del	p.Lys847del	LP	PM_supp, PS4, PM4

Truncating - Frameshift	MYBPC3	NM_000256.3	c.2591T>C	p.Phe864Serfs*15	LP	PM2_supporting y PVS1
Missense	PKP2	NM_001005242.3	c.1378G>A	p.Gly460Ser	LP	PS4, PP1_supporting, PM2_supporting, PP3
Truncating - Nonsense	TTN	NM_003319.4	c.58573C>T	p.Arg19525*	LP	PM2_supporting y PVS1
Truncating - Nonsense	TTN	NM_003319.4	c.214A>T	p.Lys72*	LP	PM2_supporting y PVS1
Truncating - Nonsense	FHL1	NM_001159700.1	c.542G>A	p.Trp181*	LP	PM2_supporting y PVS1
Missense	MYH7	NM_000257.4	c.2167C>G	p.Arg723Gly	LP	PM2_supp, PS4, PP3, PM1
Truncating - Frameshift	FLNC	NM_001458.4	c.2230_2231delTGinsA	p.Trp744Argfs*4	LP	PM2_supporting y PVS1

Truncating - Frameshift	TTN	NM_003319.4	c.74758delG	p.Glu24920Lysfs*25	LP	PM2_supporting y PVS1
Splice-site (canonical ±1/±2)	DSG2	NM_001943.3	c.-1_1delGAinsCG		LP	PM2_Supp, PVS1_moderate, PS1, PP1
Missense	MYH7	NM_000257.4	c.1618T>C	p.Phe540Leu	LP	PM2_supp, PS4_supp, PP3, PP1, PM1
Truncating - Frameshift	ALPK3			p.Ala612Glnfs*50	LP	PM2_supporting y PVS1
Missense	TTR	NM_000371.4	c.128G>A	p.Ser43Asn	LP	PM2_Supporting, PS4 y PP1_Strong
Splice-site (canonical ±1/±2)	MYBPC3	NM_000256.3	c.505+2T>A		LP	PM2_supporting y PVS1
Truncating - Frameshift	TTN	NM_003319.4	c.70360_70361insA	p.Thr23454Asnfs*8	LP	PM2_supporting y PVS1

Truncating - Nonsense	DSP	NM_004415.4	c.4699C>T	p.Gln1567*	LP	PM2_supporting y PVS1
Splice-site (canonical ±1/±2)	DMD	NM_004006.2	c.264+2_264+3insT		LP	PM2_supporting y PVS1
Truncating - Nonsense	TTN	NM_003319.4	c.9310C>T	p.Arg3104*	LP	PM2_supp, PVS1
Truncating - Nonsense	TTN	NM_003319.4	c.63895A>T	p.Lys21299*	LP	PM2_supp, PVS1
CNV (deletion)	PKD2	NM_000297.3:c.844_1094del	c.844_1094del		LP	1A, 2E, 3A
Truncating - Frameshift	TTN	NM_003319.4	c.24402delA	p.Glu8134Aspfs*7	LP	PM2_supp, PVS1
Truncating - Nonsense	TTN	NM_003319.4	c.34703_34704delTAinsAG	p.Leu11568*	LP	PM2__Supp/PVS1

Missense	TRIM63	NM_032588.3:c.68G>A	c.26393918C>T	p.Cys23Tyr	LP	PM2_supporting, PM3_Strong y PP3
Truncating - Frameshift	LMNA	NM_170707.3	c.240delC	p.Tyr81Thrfs*15	LP	PM2_supporting, PVS1.
Missense	TTR	NM_000371.4	c.277A>G	p.Ile93Val	LP	PM2_Supporting, PS4_mod, PP1_mod, PP4
Truncating - Frameshift	TTN	NM_003319.4	c.74540_74541insA	p.Asn24847Lysfs2	LP	PM2_supporting, PVS1
Truncating - Frameshift	BAG3	NM_004281.4		p.Gly197Trpfs*15	LP	PM2_supporting, PVS1
Truncating - Frameshift	DSP	NM_004415.3	c.5862_5863delGA	p.Gln1954Hisfs*2	LP	PM2_supporting, PVS1
Truncating - Nonsense	MYBPC3	NM_000256	c.2761C>T	p.Gln921*	LP	PM2_supporting, PVS1

Truncating - Nonsense	PKP2	NM_001005242.3	c.175C>T	p.Gln59*	LP	PM2_supp, PVS1
Splice-site (canonical ±1/±2)	LMNA	NM_170707.3	c.1608+1G>A		LP	PM2_supp, PVS1_strong, PP1
Truncating - Frameshift	TTN	NM_003319.4	c.57981dupC	p.Lys19328Glnfs*7	LP	PM2_supp, PVS1
Missense	MYH7	NM_000257.4	c.2555T>C	p.Met852Thr	LP	PM2_supp, PS4_mod, PM1, PP3
Truncating - Frameshift	TTN	NM_003319.4	c.57813_57816del	p.Glu19273Hisfs*9	LP	PM2_supp, PVS1
Missense	LMNA	NM_170707.4	c.1412G>A	p.Arg471His	LP	PM2_supp, PS4, PP1, PP3
Missense	MYL2	NM_000432.4	c.112C>A	p.Gln38Lys	LP	PM2_supporting, PS4_supporting, PP1_strong

Truncating - Nonsense	BAG3	NM_004281.4	c.394C>T	p.Gln132*	LP	PVS1, PM2_supporting
Missense	DES	NM_001927.4	c.46C>T	p.Arg16Cys	LP	PM2_supp, PS4_moderate, PP3, PS3_mod
Truncating - Nonsense	BAG3	NM_004281.4	c.394C>T	p.Gln132*	LP	PVS1, PM2_supporting
Truncating - Frameshift	TTN	NM_003319.4	c.11883delA	p.Gly3962Alafs*4	LP	PM2_Supporting y PVS1
Truncating - Nonsense	TTN	NM_003319.4	c.54292G>T	p.Glu18098*	LP	PM2_Supporting y PVS1
Truncating - Frameshift	TTN	NM_003319.4	c.57973delT	p.Ser19325Profs*7	LP	PM2_Supp, PVS1
Missense	TNNT2	NM_001001430.2	c.281G>T	p.Arg94Leu	LP	PM2_supp, PS4_mod, PP3, PM1, PP1

Missense	MYBPC3	NM_000256.3	c.223G>A	p.Asp75Asn	LP	PM2_supp, PS4 y PS3_supporting
Missense	TPM1	NM_001018005.2	c.841A>G	p.Met281Val	LP	PM2_supp, PS4, PP2 y PM5
Missense	FLNC	NM_001458.4	c.6902C>T	p.Pro2301Leu	LP	PM2_supp, PS4_supp, PM6, PP3_mod
Missense	TNNT2	NM_001001430.2	c.281G>T	p.Arg94Leu	LP	PM2_supp, PS4_mod, PP3, PM1, PP1
Truncating - Frameshift	MYBPC3	NM_000256.3	c.2385_2386insCCTGCACAGTACAGTGGGAGCCGCCT	p.Ala796Profs*35	LP	PM2_supp, PVS1
CNV (duplication)	PKP2	NM_004572.3	c.(1688+1_1689-1)_(2145+1_2146-1)dup		LP	1A, 2I,4L, 5D

CNV (deletion)	TTN	NM_001267550.2	c.67105_74709del		LP	PVS1*, PM2. *CNV Interpretation Scoring Metric - Copy Number LOSS. 1A, 2E: score 0.90 (LP)
In-frame deletion	DMD	NM_004006.2	c.10101_10103del	p.Glu3367del	LP	PM2, PM4, PS4_mod
Splice-site (canonical ±1/±2)	LMNA	NM_001282625	c.1158-1G>A		LP	PM2_supp, PVS1
Mitocondrial	MT-TW	NC_012920.1	m.5541C>T		LP	PS4_supp, PM2_supp, PP4, PP3, PS3_mod
Truncating - Nonsense	TRIM63	NM_032588.3	c.157C>T	p.Gln53*	LP	PM2_supp, PVS1
Splice-site (canonical ±1/±2)	TTN	NM_003319.4	c.27616+1G>A		LP	PM2_supp, PVS1
Missense	LMNA	NM_170707.3	c.370G>A	p.Glu124Lys	LP	PM2_supp, PS4, PP1_mod y PP3

Truncating - Frameshift	BAG3	NM_004281.4	c.257dup	p.Tyr86*	LP	PM2_supp, PVS1
Missense	MYBPC3	NM_000256.3	c.3065G>C	p.Arg1022Pro	LP	PS4_moderate, PP1_Moderate, PM5_supporting, PP3
In-frame deletion	MYBPC3	NM_000256.3	c.671_673delTGC	p.Leu224del	LP	PM2_supporting, PS4, PM4, PP1
Truncating - Frameshift	DSP	NM_004415.2	c.5001delG	p.Gln1667Hisfs*14	LP	PVS1, PM2_supp
Intronic affecting splicing	DSG2	NM_001943.3	c.523+4A>G		LP	PM2_supp, PS4, PP3, PS1_Supp
Splice-site (canonical ±1/±2)	MYL2	NM_000432.3	c.403-1G>C		LP	PM2_supp, PVS1_Strong, PM3
Splice-site (canonical ±1/±2)	TTN	NM_133378.4	c.47107+1G>A		LP	PM2_supp, PVS1

Missense	FLNC	NM_001458.4	c.6923C>T	p.Pro2308Leu	LP	PM2_Supporting, PS4 y PM1
Missense	MYBPC3	NM_000256.3	c.680C>T	p.Thr227Ile	LP	PM2_supporting, PS4, PP1
Missense	MYL3	NM_000258.2	c.170C>A	p.Ala57Asp	LP	PM2_supporting, PS4, PP3 y PM5
Missense	MYH7	NM_000257.3	c.5342G>A	p.Arg1781His	LP	PM2_Supporting, PS4_moderate, PP3 y PP1_moderate
Missense	TMEM43			p.Ser358Leu	P	PM2_supp, PS4, PS3, PP1_strong
Splice-site (canonical ±1/±2)	TTN	NM_003319.4:	c.17086+1G>A		P	thr
Missense	TNNT2	NM_001001430.2	c.421C>T	p.Arg141Trp	P	PM2_supp, PS4, PP3, PM6, PP1_strong, PS3_mod
Truncating - Nonsense	TTN	NM_003319.4	c.12028C>T	p.Gln4010*	P	PS4_supp, PM2_supp, PVS1

Truncating - Nonsense	DSP	NM_004415.3	c.5851C>T	p.Arg1951*	P	PM2_supp, PVS1, PS4
Missense	TMEM43	NM_024334.3	c.1073C>T	p.Ser358Leu	P	PM2_supp, PS4, PS3, PP1_strong
Truncating - Nonsense	TTN	NM_003319.4	c.12028C>T	p.Gln4010*	P	PM2_supp, PS4_supporting, PVS1
Missense	ACTC1	NM_005159.5	c.76G>A	p.Asp26Asn	P	PM2_supp, PS4, PP1_strong y PS3_supp
Missense	TTR	NM_000371.4	c.424G>A	p.Val142Ile	P	PS4, PP1, PS3_mod, PM5
Truncating - Frameshift	LAMP2	NM_002294.2	c.779_780insA	p.His260Glnfs*14	P	PM2_supp, PS4_supp, PVS1
Truncating - Frameshift	TTN	NM_003319.4	c.41800delA	p.Thr13934Profs*35	P	PM2_supp, PS4_supp, PVS1
Missense	MYBPC3	NM_000256.3	c.2308G>A	p.Asp770Asn	P	PM2_supp, PS4, PS3

Truncating - Frameshift	FLNC	NM_001458.5	c.2208delT	p.Lys737Serfs*11	P	PM2_supp, PS4_mod, PVS1
Missense	TNNT2	NM_001001430.3	c.274C>T	p.Arg92Trp	P	PM2_supp, PS4, PS3_mod, PM5, PP3
Missense	TTR	NM_000371.4	c.424G>A	p.Val142Ile	P	PS4, PP1, PS3_mod, PM5
Missense	TTR	NM_000371.4	c.424G>A	p.Val142Ile	P	PS4, PP1, PS3_mod, PM5
Missense	MYH7	NM_000257.4	c.2156G>A	p.Arg719Gln	P	PM2_supp, PS4_mod, PP3, PS3_supp, PM1, PP1, PM6
Splice-site (canonical ±1/±2)	TTN	NM_003319.4	c.26995+1G>A		P	PM2_supp, PS4_mod, y PVS1
Missense	LMNA	NM_170707.3	c.569G>A	p.Arg190Gln	P	PM2_supporting, PS4, PP1_moderated, PM5, PS3_supporting

Truncating - Nonsense	LMNA	NM_170707.3	c.1401G>A	p.Trp467*	P	PM2_supp, PVS1, PS4_mod
Splice-site (canonical ±1/±2)	DMD	NM_004006.3	c.2169-2A>T		P	PM2_supp, PVS1, PS4_supp
Missense	MYH7	NM_000257.3	c.2146G>A	p.Gly716Arg	P	PM2_supporting, PS4, PP1, PP3, PS3, PM1
Splice-site (canonical ±1/±2)	MYBPC3	NM_000256.3	c.2149-1G>A		P	PM2_supp, PS4, PP1_Strong, PVS1 y PS1
In-frame deletion	TTR	NM_00371.3	c.424_426del	p.Val142del	P	PM2_Supporting, PS4 y PP1_strong
Missense	TTR	NM_000371.4	c.424G>A	p.Val142Ile	P	PS4, PP1, PS3_mod, PM5
Missense	RBM20	NM_001134363	c.1900C>T	p.Arg634Trp	P	PM2_supp, PP1, PP2, PM5, PS4, PP3
Missense	MYH7	NM_000257.2	c.2389G>A	p.Ala797Thr	P	PS4, PP1_strong y PM5

Truncating - Frameshift	DSP	NM_004415.2	c.2131_2132delAG	p.Ser711Cysfs*4	P	PM2_supp, PS4_mod, PVS1, PP1
CNV (deletion)	DMD	NM_004006.2	c.(6912+1_6913-1)_(7872+1_7873-1)del;	Del ex. 48_53	P	1A, 2E, 3A, 5G
Missense	MYH7	NM_000257.3	c.2674C>A	MYH7 p.Gln892Lys	P	PM2_supp, PM1, PP3, PS4, PP1_strong
Splice-site (canonical ±1/±2)	MYBPC3	NM_000256.3	c2308+1G>A		P	PM2_supp, PVS1, PS4_supp, PP1
Truncating - Frameshift	BAG3	NM_004281.3	c.382_383insAG	p.Ala128Glufs*84	P	PVS1, PM2_supp, PS4_supp
Missense	LMNA	NM_170707.3	c.1003C>T	p.Arg335Trp	P	PM2_supp, PS4_mod, PP1_strong, PS3_mod, PM5_supp, PP3

Missense	LAMP2	NM_002294.2	c.928G>A	p.Val310Ile	P	PM2_supp, PVS1, PS4_mod, PP1, PM6
Missense	MYH7	NM_000257.2	c.216C>G	p..Arg723Gly	P	PM2_supp, PS4, PM1, PP1_strong, PP3, PS3_supp
Missense	MYH7	NM_000257.3	c.2389G>A	p.Ala797Thr	P	PS4, PP1_Strong y PM5
Truncating - Nonsense	FLNC	NM_001458.5	c.5398G>T	p.Gly1800*	P	PM2_supp, PVS1, PS4_mod, PP1
Truncating - Nonsense	TTN	NM_003319.4	c.20299C>T	p.Arg6767*	P	PM2_supp, PVS1, PS4_supp
Missense	MYH7	NM_000257.2	c.1357C>T	p.Arg453Cys	P	PM2_supp, PS4, PM6, PP1_mod, PP3, PM1, PS3_supp
Missense	TNNT2	NM_001001430.2	c.275G>A	p.Arg92Gln	P	PM2_supp, PS4, PP1_strong, PM5, PS3_supp

In-frame deletion	MYBPC3	NM_000256.3	c.1513_1515del	p.Lys505del	P	PM2_supp, PS4, PP1_mod, PM4 y PS3_supp
Truncating - Nonsense	FLNC	NM_001458	c.1948C>T	p.Arg650*	P	PM2_supp, PS4_mod, PVS1
Truncating - Nonsense	TTN	NM_003319.4	c.74032C>T	p.Arg24678*	P	PM2_supp, PVS1, PS4_supp, PP1
Missense	MYH7	NM_000257.4	c.2167C>G	p.Arg723Gly	P	PM2_supporting, PS4, PP1_strong, PM5, and PS3_supporting
Missense	TPM1	NM_001018005	c.688G>A	p.Asp230Asn	P	PM2_supp, PS4_mod, PP2, PS3_mod,PP1
Splice-site (canonical ±1/±2)	MYBPC3	NM_000256	c.1928-2A>G		P	PM2_supp, PS4, PP1, and PVS1
In-frame deletion	TTR	NM_000371.4	c.424_426del	p.Val142del	P	PM2_supporting, PS4 y PP1_strong

Splice-site (canonical ±1/±2)	MYBPC3	NM_000256.3	c.2308+1G>A		P	PM2_supp PS4, PP1, PVS1, PS3 y PS1_supp
Missense	PRKAG2	NM_016203	c.905G>A	p.Arg302Gln	P	PM2_supp, PS4, PP1, PS3, PS2
Truncating - Nonsense	FLNC	NM_001458.5	c.4021C>T	p.Arg1341*	P	PM2_supp, PS4_supp, PVS1
Truncating - Nonsense	LAMP2	NM_002294.3	c.877C>T	p.Arg293*	P	PM2_supp, PS4_mod, PVS1
In-frame deletion	FHOD3	NM_001281740.2	c.1580_1582delCCT	p.Ser527del	P	PM2_supp, PS4, PP1_strong y PM4
Missense	MYL3	NM_000258.2	c.427G>A	p.Glu143Lys	P	PM2_supp, PS4, PS3, PP1
Missense	LMNA	NM_170707.4	c.568C>T	p.Arg190Trp	P	PM2_supp, PS4, PP1_moderate, PS3_supporting y PM5.
Missense	MYH7	NM_000257.4	c.1208G>A	p.Arg403Gln	P	PM2_supp, PS4, PM5, PS3 y PP1

In-frame deletion	TNNT2	NM_001001430.3	c.487_489del	p.Glu163del	P	PS4, PM4, PM1, PP1, PS3
Missense	TNNT2	NM_001001430.3	c.275G>A	p.Arg92Gln	P	PM2_supp, PS4, PP1_Strong, PM5, PS3_supporting
Truncating - Nonsense	MYBPC3	NM_000256.3	c.787G>T	p.Gly263*	P	PM2_supp, PS4, PVS1 y PP1
Splice-site (canonical ±1/±2)	PKP2	NM_004572.3	c.2146-1G>C		P	PS4, PVS1, PP1
Truncating - Nonsense	BAG3	NM_004281	c.262C>T	p.Gln88*	P	PM2_supp, PVS1, PP1
Truncating - Nonsense	TTN	NM_003319.4	c.20497C>T	p.Arg6833*	P	PM2_supp, PS4_supp, PVS1
Missense	MYH7	NM_000257	c.1357C>T	p.Arg453Gly	P	PM2_supp, PS4, PP1, PS3_mod, PM1 y PP3
Missense	MYH7	NM_000257.3	c.1208G>A	p.Arg403Gln	P	PM2_supp, PS4, PM5, PS3 y PP1

Missense	ACTC1	NM_005159.5	c.268C>T	p.His90Tys	P	PM2_supp, PS4, PP1_strong, PP3
Missense	MYBPC3	NM_000256.3	c.772G>A	p.Glu258Lys	P	PM2_supp, PM5, PP3, PS4_supp, PP4
CNV (deletion)	DMD	NM_004006.2	Delección exones 45 al 55 del		P	1A 2E 3A 5G
Missense	MYH7	NM_000257.4	c.2156G>A	p.Arg719Gln	P	PM2_supp, PS4, PM5, PP3, PS3_mod y PP1.
CNV (deletion)	DMD	NM_004006.2:c.6913_7542del	c.6439_7872del		P	1A, 2E, 3A, 5H ver nomebnclatura
In-frame deletion	MYBPC3	NM_000256.3	c.1513_1515delAAG	p.Lys505del	P	PM2_supp, PS4, PP1_mod, PM4 y PS3_supp.
Missense	MYBPC3	NM_000256.3	c.646G>A	p.Ala216Thr	P	PS4, PP1 y PVS1
Truncating - Frameshift	PKP2	NM_004572.3	c.987delT	p.Ser329Argfs*23	P	PVS1, PM2_supp, PS4, PP1_mod

Truncating - Nonsense	BAG3	NM_004281.3	c.457C>T	p.Gln153*	P	PM2_supporting, PVS1 y PP1
Truncating - Nonsense	DSC2	NM_024422.6	c.1123C>T	p.Arg375*	P	PM2_supp, PS4_mod, PVS1
Missense	DSG2	NM_001943.3	c.1003A>G	p.Thr335Ala	P	PS4_supp, PM3_Strong, PP1_strong, PP3_mod
Truncating - Nonsense	TTN	NM_003319.4	c.11428G>T	p.Glu3810*	P	PM2_Supp, PS4_Mod, PP1 y PVS1
Missense	LMNA	NM_170707.3	c.1130G>A	p.Arg377His	P	PM2_supp, PS4, PP1, PP3, PS3, PM5
Truncating - Frameshift	TTN	NM_003319.4	c.8929delC	p.His2977Thrfs*3	P	PM2_supp, PS4_supp y PVS1
CNV (deletion)	DMD	NM_004006.2	c.6913_7200del	Del exon 48_49, g.(?_31854837)_(31893492_?)del	P	1A, 2E, 3A, 4L, 5H.

Missense	MYH7	NM_000257.4	c.2207T>C	p.Ile736Thr	P	PM2_supp, PS4, PP1_mod, PP3 y PM5
Truncating - Nonsense	LMNA	NM_170707.4	c.961C>T	p.Arg321*	P	PM2_supp, PVS1, PS4_mod, PP1
Splice-site (canonical ±1/±2)	MYBPC3	NM_000256.3	c.2308+1G>A		P	PM2_supp, PS4, PP1, PVS1, PS3 y PS1_supp
Truncating - Frameshift	MYBPC3	NM_000256.3	c.1800del	p.Lys600Asnfs*2	P	PM2_supp, PS4, PP1 y PVS1
Missense	LMNA	NM_170707.3	c.1130G>A	p.Arg377His	P	PM2_supp, PS4, PP1_mod, PM5, PS3_supp, PP3
Missense	MYH7	NM_000257.4	c.1816G>A	p.Val606Met	P	PM2_supp, PS4, PP1, PP3, PS3, PM5
Truncating - Nonsense	TTN	NM_133378	c.44305C>T	p.Arg14769*	P	PM2_supp, PS4_supp y PVS1

Missense	MYH7	NM_000257.4	c.2389G>A	p.Ala797Thr	P	PS4, PP1_Strong y PM5
In-frame deletion	PLN	NM_002667.4	c.40_42delAGA	p.Arg14del	P	PM2_supp, PS4, PM4 y PS3
Missense	ACTC1	NM_005159.5	c.301G>A	p.Glu101Lys	P	PS4, PM2_supp, PP1 y PS3_mod
Truncating - Nonsense	TTN	NM_003319.4	c.11428G>T	p.Glu3810*	P	PM2_supp, PVS1, PS4_mod, PP1
Missense	MYH7	NM_000257.4	c.2701G>C	p.Ala901Pro	P	PM2_supp, PS4_mod, PP3, PM1
Truncating - Frameshift	MYBPC3	NM_000256.3	c.1800del	p.Lys600Asnfs*2	P	PM2_supp, PS4, PP1 y PVS1
Truncating - Nonsense	MYBPC3	NM_000256.3	c.2375G>A	p.Trp792*	P	PM2_supp, PS4_supp, y PVS1
CNV (deletion)	DMD	NM_004006.2	c.6913_7542del	p.Val2305_Lys2514del	P	1A, 2E, 3A, 4L

Truncating - Frameshift	MYBPC3	NM_000256.3	c.1800del	p.Lys600Asnfs*2	P	PM2_supp, PS4, PP1 y PVS1
Truncating - Nonsense	TTN	NM_003319.4	c.24814C>T	p.Arg8272*	P	PM2_Supp, PS4_Supp y PVS1
Missense	ACTC1	NM_005159.4	c.301G>A	p.Glu101Lys	P	PS4, PM2_supp, PP1 y PS3_mod
Truncating - Frameshift	PKP2	NM_001005242.3	c.987del	p.Ser329Argfs*23	P	PM2_supp, PS4, PP1_mod, PVS1
Truncating - Nonsense	TTN	NM_003319.4	c.37258C>T	p.Arg12420*	P	PM2_supporting, PS4 y PVS1
Missense	DES	NM_001927.4	c.1360C>T	p.Arg454Trp	P	PM2_supporting, PS4, PS3, PM6, PP1, PP3
Missense	MYH7	NM_000257.4	c.2156G>A	p.Arg719Gln	P	PM2_supp, PS4_mod, PP3, PS3_supp, PM1, PP1, PM6

Truncating - Nonsense	TTN	NM_003319.4	c.53521C>T	p.Arg17841*	P	PVS1, PM2_suppp, PS4
Missense	TPM1	NM_001018005.2	c.62G>T	p.Arg21Leu	P	PM2_Supporting, PS4, PP1_Strong, PP3 y PM5_Supporting PP2.
Missense	LMNA	NM_170707.4	c.568C>T	p.Arg190Trp	P	PM2_supporting, PS4, PP1_moderate, PS3_supporting y PM5
Missense	TNNI3	NM_000363.5	c.470C>T	p.Ala157Val	P	PM2_Supporting, PS4, PP1_Strong, PS3_Supporting y PM1.
Missense	MYH7	NM_000257.4	c.2389G>A	p.Ala797Thr	P	PS4, PP1_Strong y PM5
Missense	MYH7	NM_000257.4	c.2156G>A	p.Arg719Gln	P	PM2_supp, PS4, PM5, PP3, PS3_mod y PP1.

Mitocondrial	MT-TL1	m3243A>G-MTL1	m3243A>G-MTL1	m3243A>G-MTL1	P	PM2_supp, PS4, PS3, PP3, y PP1
Truncating - Nonsense	TTN	NM_003319.4	c.56221C>T	p.Arg18741*	P	PM2_supp, PS4_mod, PVS1
Truncating - Frameshift	TTN	NM_003319.4	c.12013_12016delAAAG	p.Lys4005Cysfs*25	P	PM2_supporting, PVS1, PP1
Missense	MYH7	NM_000257.4	c.1063G>A	p.Ala355Thr	P	PS4, PP1, PM1, PP2, PM2
In-frame deletion	TTR	NM_000371.4	c.424_426del	p.Val142del	P	PM2_Supporting, PS4 y PP1_Strong
Splice-site (canonical ±1/±2)	MYBPC3	NM_000256.3	c.2149-1G>A		P	PM2_Supporting, PS4, PP1_Strong, PVS1 y PS1
Splice-site (canonical ±1/±2)	DMD	NM_004006.2	c.31+1G>T		P	PM2_supp, PVS1, PS4_mod, PP1

Splice-site (canonical ±1/±2)	MYBPC3	NM_000256.3	c.2150-1G>A		P	PM2_Supporting, PS4, PP1_Strong, PVS1 y PS1
Truncating - Frameshift	DSG2	NM_001943.3	c.485A>T	p.Asp162Valfs*10	P	PM2_supporting, PS4_supp y PVS1
Truncating - Frameshift	TBX20	NM_001077653.2	c.675delT	p.His225Glnfs*22	P	PVS1, PP1_Strong, PM2, PS4_Supporting
Truncating - Nonsense	TTN	NM_003319.4	c.37258C>T	p.Arg12420*	P	PM2_supporting, PS4 y PVS1.
Splice-site (canonical ±1/±2)	MYBPC3	NM_000256.3	c.2308+1G>A		P	PM2_supporting, PS4, PP1_strong, PVS1, PS3
Missense	TTR	NM_000371.4	c.128G>A	p.Ser43Asn	P	PM2_Supporting, PS4 y PP1_Strong
Missense	MYH7	NM_000257.4	c.2156G>A	p.Arg719Gln	P	PM2_supp, PS4, PM5, PP3, PS3_mod y PP1.

Missense	MYH7	NM_000257.4	c.1357C>T	p.Arg453Cys	P	PM2_supp, PS4, PP1, PS3_mod, PM1 y PP3
Missense	RBM20	NM_001134363.3	c.1913C>T	p.Pro638Leu	P	PM2_supp, PS4, PS3, PM1, PP1.
Missense	DSG2	NM_001943.4	c.1003A>G	p.Thr335Ala	P	PS4_supporting/PM3_Strong, PP1_strong, PP3_moderate.
Truncating - Nonsense	PKP2	NM_004572.3	c.2293G>T	p.Glu765*	P	PS4_moderate, PM2_supporting, PVS1
CNV (deletion)	DMD	NM_004006.2	?	Delección del exón 47 al 52	P	1A, 2E, 3A, 5G
Splice-site (canonical ±1/±2)	LMNA	NM_170707.3	c.1158-1G>A		P	PM2_supp, PVS1, PP1
Missense	LMNA	NM_170707.3	c.568C>T	p.Arg190Trp	P	PM2_supporting, PS4, PP1_moderate, PS3_supporting y PM5

Truncating - Nonsense	TTN	NM_133378.4	c.43732C>T	p.Gln14578*	P	PM2_supporting, PS4_supporting, PVS1.
Truncating - Nonsense	TTN	NM_003319	c.37258C>T	p.Arg12420*	P	PM2_supporting, PS4 y PVS1
Splice-site (canonical ±1/±2)	MYBPC3	NM_000256.3	c.1351+1G>A		P	PM2_supporting, PS4_moderate y PVS1
Splice-site (canonical ±1/±2)	LMNA	NM_170707.3	c.1158-1G>A		P	PM2_Supporting, PS4_mod, PP1_mod, PP4
Missense	DES	NM_001927.3	c.1203G>C	p.Glu401Asp	P	PM2_supporting, PP1_Strong, PM5 y PS3
Missense	TNNT2	NM_001001430	c.274C>T	p.Arg92Trp	P	PM2_supporting, PS4, PP1, PS3_moderate y PM5
Missense	LMNA	NM_170707.3	c.568C>T	p.Arg190Trp	P	PM2_supporting, PS4, PP1_moderate,

						PS3_supporting y PM5
Missense	PRKAG2	NM_016203	c.905G>A	p.Arg302Gln	P	PM2_supporting, PS4, PP1, PS3, PS2
Missense	LMNA	NM_170707	c.568C>T	p.Arg190Trp	P	PM2_supporting, PS4, PP1_moderate, PS3_supporting y PM5
In-frame deletion	TNNT2	NM_001276345.2	c.508GA>G	p.Glu163del	P	PM2_supporting, PS4, PM4, PM1, PP1, PS3.
Splice-site (canonical ±1/±2)	GLA	NM_000169.2	c.639+1G>A		P	PS4_supp, PVS1, PM2_supp
Missense	MYBPC3	NM_000256.3	c.1483C>T	p.Arg495Trp	P	PS4, PM5, PP1_Moderate y PP3
Missense	LMNA	NM_170707.4	c.1130G>A	p.Arg377His	P	PM2_supp, PS4, PP1_mod, PM5, PS3_supp, PP3

Truncating - Frameshift	TTN	NM_003319.4	c.21796_21797insATCA	p.Ile7266Asnfs*3	P	PM2_supporting, PS4_supporting y PVS1.
Missense	MYBPC3	NM_000256	c.1505G>A	p.Arg502Gln	P	PS4, PP1_moderate, PM2_supporting, PP3 y PM5.
Missense	LMNA	NM_170707.4	c.1130G>A	p.Arg377His	P	PM2_supp, PS4, PP1_mod, PM5, PS3_supp, PP3
Truncating - Nonsense	BAG3	NM_004281.3:	c.367C>T	p.Arg123*	P	PS4_moderate, PM2_supporting, PVS1
Missense	LMNA	NM_170707.4	c.481G>A	p.Glu161Lys	P	PM2_supp, PS4, PP1_Strong, PS3_supp
Missense	MYH7	NM_000257.4	c.746G>A	p.Arg249Gln	P	PM2_supp, PS4, PS2, PP1_strong, PM1, PS3_supporting
Splice-site (canonical ±1/±2)	MYBPC3	NM_000256	c.1351+1G>A		P	PM2_supp, PS4_moderate y PVS1.

Missense	MYBPC3	NM_000253.3	c.1484G>A	p.Arg495Gln	P	PM2_supp, PS4_mod, PS3, PM5, PP1, PP3
Missense	DSG2	NM_001943.4	c.146G>A	p.Arg49His	P	PM2_supp, PS4, PP1, PS3, PS2, PM5
Missense	MYH7	NM_000257.3	c.2674C>A	p.Gln892Lys	P	PM2_supp, PM1, PP3, PS4, PP1_strong
Missense	RBM20	NM_001134363.3	c.1907G>A	p.Arg636His	P	PS4, PP1, PM2, PM5, PM1, PP3
Truncating - Frameshift	TTN	NM_003319.4	c.43951_43961del	p.Gly14651Hisfs*6	P	PM2_supp, PS4, PVS1
Missense	TNNI3	NM_000363.4	c.485G>A	p.Arg162Gln	P	PM2_supp, PS4, PS3_supp, PP1_strong , PM5.
Missense	MYH7	NM_000257.4	c.2167C>G	p.Arg723Gly	P	PM2_supp, PS4, PP1_Strong, PM5, and PS3_Supporting

Missense	TNNI3	NM_000363.4	c.434G>A	p.Arg145Gln	P	PM2_supp, PS4, PP1_supporting, PS3_supporting y PM5.
Truncating - Nonsense	MYBPC3	NM_000256.3	c.1522C_T	p.Gln508*	P	PM2_supp, PS4_supp, PVS1
Splice-site (canonical ±1/±2)	MYBPC3	NM_000256.3	c.2308+1G>A		P	PM2_Supporting, PS4, PP1, PVS1 y PS1_Supporting
Missense	MYBPC3	NM_000256.3	c.772G>A	p.Glu258Lys	P	PS4, PP1 y PVS1.
Splice-site (canonical ±1/±2)	MYBPC3	NM_000256.3	c.2308+1G>A		P	PM2_Supporting, PS4, PP1, PVS1 y PS1_Supporting
Truncating - Nonsense	TTN	NM_003319.4	c.58072C>T	p.Arg19358*	P	PM2_supporting, PS4, PVS1
In-frame deletion	PLN	NM_002667.5	c.40_42del	p.Argdel	P	PM2_supporting, PS4, PM4 y PS3

Splice-site (canonical ±1/±2)	MYBPC3	NM_000256.3	IVS23+1 G>A		P	PM2_Supporting, PS4, PP1, PVS1 y PS1_Supporting
Missense	MYH7	NM_000257	c.2146G>A	Gly716Arg	P	PM2_supporting, PS4, PP1, PP3, PS3, PM1
Truncating - Frameshift	MYBPC3	NM_000256.3	c.2670dup	p.Arg891Alafs*160	P	PM2_Supporting, PS4, PP1 y PVS1.
Missense	LMNA	NM_170707	c.1129C>T	Arg377Cys	P	PM2_supporting, PS4, PP1, PP3 y PM5
Truncating - Frameshift	MYBPC3	NM_000256.3	c.2670dup	p.Arg891Alafs*160	P	PM2_Supporting, PS4, PP1 y PVS1.
Missense	RBM20	NM_001134363.3	c.1907G>A	p.Arg636His	P	PM2_supporting, PS4, PP1_strong, PP3, PM5_strong
In-frame deletion	PLN	NM_002667.4	c.40_42delAGA	p.Arg14del	P	PM2_supp, PS4, PM4 y PS3

Truncating - Nonsense	MYBPC3	NM_000256.3	c.2512G>T	p.Glu838*	P	PM2_supporting, PS4_supporting, PVS1
Missense	MYH7	NM_000257.4	c.4130C>T	p.Thr1377Met	P	PM2_Supporting, PS4, PP3 y PP1_Strong
Missense	FHOD3	NM_001281740.3	c.1583A>G	p.Tyr528Cys	P	PM2_Supporting, PS4, PP1_Strong
Missense	MYH7	NM_000257.4	c.1357C>T	p.Arg453Cys	P	PM2_supporting, PS4, PP1, PS3
Missense	TNNT2	NM_001001430.2	c.518G>A	p.Arg173Gln	P	PS4_mod, PP1_strong, PM1, PM2,
In-frame deletion	TNNT2	NM_001001430.2	c.629_631delAGA	p.Lys210del	P	PM2_supporting, PS4, PP1, PM4, PS3_supporting.
Missense	MYBPC3	NM_000256.3	c.772G>A	p.Glu258Lys	P	PS4, PP1 y PVS1.
Splice-site (canonical ±1/±2)	MYBPC3	NM_000256.3	c.821+1G>A		P	PM2_Supporting, PS4, PP1, PS1_Supporting y PVS1

Missense	TNNT2	NM_001001430.2	c.275G>A	p.Arg92Gln	P	PM2_supporting, PS4, PP1_Strong, PM5, PS3_mod
Truncating - Frameshift	FLNC	NM_001458.5	c.2208delT	p.Lys737Serfs*11	P	PM2_supporting, PS4_moderate y PVS1.
Missense	TTR	NM_000371.4	c.424G>A	p.Val142Ile	P	PS4, PP1_supporting, PS3_moderate, and PM5
Truncating - Frameshift	FLNC	NM_000257.4	c.2208delT	p.Lys737Serfs*11.	P	PM2_sup, PS4_moderate y PVS1
Missense	TNNT2	NM_001001430.2	c.275G>A	p.Arg92Gln	P	PM2_supporting, PS4, PP1_Strong, PM5, PS3_mod
Missense	MYH7	NM_000257.4	c.1012G>A	p.Val338Met	P	PM2_supp, PS4, PM1, PP3, PP1
Truncating - Frameshift	TTN	NM_003319.4	c.41800delA	p.Thr13934Profs*35	P	PS4_supporting, PM2_supporting, PVS1

Missense	DES	NM_001927.3	c.1216C>T	p.Arg406Trp	P	PM2_supp, PS4, PM5, PS3_supp, PP1 y PS2
Missense	EMD	NM_000117.2	c.77T>C	p.Val26Ala	P	PM2_supp, PS4, PP1_strong y BP4
Splice-site (canonical ±1/±2)	MYBPC3	NM_000256.3	c.2603-2A>G		P	PM2_supp, PVS1, PS4
Splice-site (canonical ±1/±2)	MYBPC3	NM_000256.3	c.1351+1G>A		P	PM2_supp, PVS1, PS4_mod,
Missense	FHL1	NM_001159700.1	c.764G>C	p.Cys255Ser	P	PM2_Supporting, PS4_Moderate, PP1_Strong, PM5, PP3
Missense	RBM20	NM_001134363.3	c.1901G>A	p.Arg634Gln	P	PM2_supp, PM5, PS4, PP1, PP3, PP2, PS3
Truncating - Frameshift	PKP2	NM_004572.3	c.775_776insG	p.Glu259Glyfs*77	P	PM2_supporting, PVS1 y PS4

Truncating - Nonsense	TTN	NM_003319.4	c.48240G>A	p.Trp16080*	P	PM2_supporting, PS4_moderate y PVS1
Truncating - Nonsense	TTN	NM_003319.4	c.24826C>T	p.Arg8276*	P	PM2_supp, PS4_Moderate y PVS1
Truncating - Frameshift	PKD2	NM_000297.3	c.1194_1195delAA	p.Glu400Glyfs*8	P	PVS1, PM2_supp, PS4_supp
Truncating - Frameshift	TTN	NM_003319.4	c.17887delG		P	PM2_Supp, PS4_Supp y PVS1
Truncating - Frameshift	TRIM63	NM_032588.3	c.481_482del	p.Ser161Cysfs*8	P	PM2_Supporting, PM3_Strong y PVS1_Strong
Missense	DSG2	NM_001943.3	c.1003A>G	p.Thr335Ala	P	PM3_Strong, PP1_strong, PP3_moderate
Missense	MYBPC3	NM_000256.3	c.1483C>G	p.Arg495Gly	P	PS4, PM2_suporting, PM5, PS3_sup y PP1_mod

Missense	MYBPC3	NM_000256.3	c.3676C>T	p.Arg1226Cys	RF	PM2_supporting, PS4_Moderate
Missense	MYBPC3	NM_000256.3	c.1321G>A	p.Glu441Lys	RF	
Expansión tripletes	DMPK			CTG 70-80 repeats		
Intronic affecting splicing	MYL2		c.311G>T + 403-1G>C + IVST7			LP
Missense	KCNE1			p.Ala389Thr	RF	
Missense	TCAP	NM_003673.3	c.472C>G	p.Arg158Gly	VUS	PM2_supp, PP3
Missense	DMD	NM_004006.2	c.9907G>C	p.Ala3303Pro	VUS	PM2_supp, PP3
Missense	PKD1	NM_001009944.2	c.9559G>A	p.Asp3187Asn	VUS	PM2_supp, PP3
Missense	LMNA	NM_170707.3	c.1234G>C	p.Gly412Arg	VUS	PM2_supp
Missense	RBM20	NM_001134363.2:	c.1139G>A	p.Arg380Gln	VUS	BP4

Missense	TNNT2	NM_001001430.2	c.178A>G	p.Met60Val	VUS	PM2_supp, BP4
Missense	ACTC1	NM_005159.4	c.532A>T	p.Asp181His	VUS	PM2_supp, PP3
Mitocondrial	TIMM10	NM_012456.2	c.190C>G	p.R64G	VUS	PM2_supp
Intronic affecting splicing	FLNC	NM_001458.4	c.4128-9A>C		VUS	PM2_supp, BP4
Missense	MYH7	NM_000257.3	c.5741A>T	pGlu1914Val	VUS	PM2_Sup, PP3, PM5_Supp
Missense	VCL	NM_014000.2	c.2378C>A	p.Thr793Asn	VUS	PM2_supp, PP3
Missense	DSP	NM_004415.3	c.8393C>G	p.Thr2798Ser	VUS	PM2_supp, BP4
Missense	TTN	NM_003319.4	c.17806A>C	p.Asn5936His	VUS	PM2_supp, BP4, BP1
Truncating - Frameshift	FLNC	NM_001458.5	c.8178A>G	p.(*2726Trpext*47)	VUS	PM2_supp, PM4

Splice-site (canonical ±1/±2)	DSP	NM_004415.4	c.777+2T>C		VUS	PM2_supp, PVS1_mod
Missense	FLNC	NM_001458.5	c.140A>G	p.Asn47Ser	VUS	PM2_supp, PP3
Missense	MYH7	NM_000257.4	c.706G>A	p.Val236Ile	VUS	PM2_supp, PM1 y BP5
Missense	DMD	NM_004006.2	c.9625G>C	p.Ala3209Pro	VUS	PM2_supp
Missense	DMD	NM_004006.2	c.3103C>A	p.Gln1035Lys	VUS	PM2_supp, BP4
Missense	RYR2	NM_001035	c.12440G>C	p.Arg4147Thr	VUS	PM2_supp, PP3
Missense	DMD	NM_004006.3	c.9395C>A	p.Ala3132Asp	VUS	PM2_supp , PS4_supp
Missense	FHL2	NM_001450	c.523A>C	p.Thr175Pro	VUS	PP3
Missense	FLNC	NM_001458	c.3872G>A	p.Arg1291His	VUS	PM2_supp
	DSP	NM_004415.3	c.3134G>A	p.Arg1045Gln	VUS	PM2_supp, PS4_supp

	ABCC9	NM_005691.3	c.2331C>G	p.Asn777Lys	VUS	PM2_supp
Missense	FKTN	NM_006731.2	c.875G>C	p.Gly292Ala	VUS	PM2_supp, PP3
Missense	FLNC	NM_001458.4	c.1216G>A	p.Gly406Ser	VUS	PM2_supp, PP3
Intronic affecting splicing	FLNC	NM_001458	c.6485-3C>G	IVS39-3C>G	VUS	PM2_supp, PP3, PS1
Missense	LMNA		c.1364G>A	p.Arg455His	VUS	PM2_supp
Missense	TNNI3	NM_000363	c.140T>C	p.Leu47Ser	VUS	PM2_supp, PP3
Missense	MYBPC3	NM_000256	c.817C>T	p.Arg273Cys	VUS	PM2_supp
Missense	PKP2	NM_00457.3	c.1244T>G	p.Val415Gly	VUS	PM2_supp, BP4
Missense	FLNC	NM_001458.5	c.6802G>A	p.Glu2268Lys	VUS	PM2_supp
Missense	TNNI3K	NM_015978.2	c.845A>G	p.Lys282Arg	VUS	

Missense	ACTA1	NM_001100.3	c.787C>G	p.Leu263Val	VUS	PM2_supp, PP3
Missense	FLNC	NM_001458.4	c.4970G>T	p.Arg1657Leu	VUS	PM2_supp
Missense	FLNC	NM_001458.4:	c.1568T>C	p.Val523Ala	VUS	PP3
Missense	DSG2	NM_001943.3	c.2437T>C	p.Cys813Arg	VUS	PM2_supp, PP3
Missense	MYBPC3	NM_000256	c.817C>T	p.Arg273Cys	VUS	PM2_supp
Intronic affecting splicing	DMD	NM_004006.2	c.6118-15A>G		VUS	PM2_supp, PP3, PP1
	RYR2	NM_001035.2	c.13718G>A	p.Arg4573His	VUS	PP3
Missense	FLNC	NM_001458	c.3190A>G	p.Lys1064Glu	VUS	PM2_supp, PP3
Missense	SYNE1	NM_182961.3	c.25115G>T	p.Gly8372Val	VUS	PM2_supp
Missense	MYPN	NM_001256268	c.2242C>T	p.Arg748Cys	VUS	PM2_supp

Splice-site (non-canonical intronic)	RBM20	NM_001134363.2:c.3574-6C>G			VUS	PM2_supp
Missense	PKP2	NM_004572.3	c.2464A>G	p.Ile822Val	VUS	PM2_supp, BP4
Missense	DSC2	NM_024422.6	c.2603C>T	p.Ser868Phe	VUS	PM2_supp
Missense	ANK2	NM_001148.6	c.11813A>G	p.Lys3938Arg	VUS	PM2_supp, BP4
Missense	PKP2	NM_004572.3	c.14G>A	p.Gly5Asp	VUS	PM2_supp
Missense	KCNQ1	NM_000218.2	c.859G>A	p.Ala287Thr	VUS	PM2_supp, PM1, PP3
Missense	DSC2	NM_024422.4	c.1165T>A	p.Trp389Arg	VUS	PM2_supp, PP3
Missense	JUP	NM_021991.2	c.107C>T	p.Ser36Phe	VUS	PM2_Supp
Missense	MYBPC3	NM_000256.3	c.1039G>A	p.Gly347Ser	VUS	PM2_supp
Missense	SYNE1	NM_182961.3	c.1879G>T	p.Ala627Ser	VUS	PM2_supp

Missense	JPH2	NM_020433.4	c.38C>T	p.Ala13Val	VUS	PM2_supp, BP4
Missense	JUP	NM_021991.2	c.107C>T	p.T19I	VUS	PM2_supp
Missense	MYBPC3	NM_000256.3	c.2672G>A	p.Arg891Gln	VUS	PM2_supp
Missense	MYL2	NM_000432.4	c.103A>G	p.Ile35Val	VUS	PM2_supp, PP3, PP1
Missense	ACTN2	NM_001103.4	c.1250C>T	p.Ala417Val	VUS	PM2_supp, BP4
Missense	CASQ2	NM_001232.3	c.1046A>T	p.Asp349Val	VUS	PM2_supp
Missense	MYBPC3	NP_000247.2	c.1607C>A	p.Ala536Asp	VUS	PM2_supp, PP3
Missense	NKX2-5	NM_004387.3	c.178G>C	p.Glu60Gln	VUS	PM2_supp, BP4
Missense	MYH7	NM_000257.4	c.2459_2460delCCinsAA	p.Ala820Glu	VUS	PM2_supp, PM1

Missense	MYL3	NM_000258.3	c.197A>C	p.Lys66Thr	VUS	PM2_supp
Missense	RBM20	NM_001134363.2	c.3242C>T	Pro1081Leu	VUS	PM2_supp, BP4
Missense	FLNC	NM_001458.5	c.7000G>A	p.Glu2334Lys	VUS	PM2_supp, PS4_supp, PP3
Splice-site (non-canonical intronic)	TTN	NM_003319.4	c.15891+52G>A		VUS	PM2_supp, BP4
Truncating - Nonsense	DTNA	NM_001390.4	c.769C>T	p.Arg257*	VUS	PM2_supp, PP3
Sinonima	FLNC	NM_001458.4	c.5163G>A	p.Gly1721=	VUS	PM2_supporting y PP3
Missense	SCN5A	NM_198056.2	c.1446G>A	p.Met482Ile	VUS	PM2_supp
Intronica	TTN	NM_003319.4	c.13282+42117del	p.Glu10052Aspfs*25	VUS	PM2_supp
Delins	BAG3	NM_004281.3	c.89_91delTCGinsACC	p.Ile30_Asp31delinsAsnHis	VUS	PM2_supp, PS4_supp, PP1

Truncating - Nonsense	LDB3	NM_007078.2	c.655C>T	p.Arg219*	VUS	PM2_supporting
Splice-site (non-canonical intronic)	DMD	NM_004006.2	c.6615-10_6615-8delCTT		VUS	PM2_supporting
Missense	LMNA	NC_000001.10	c.886C>T	p.Arg296Cys	VUS	PM2_supporting, PP3
Sinonima	ACTC1	NM_005159.4	c456C>A	p.gly152=	VUS	PM2_supporting
Missense	ACTN2	NM_001103.3	c.559T>C	p.Cys187Arg	VUS	PM2_supporting, PP3
Missense	FXN	NM_000144.4	c.53C>T	p.Pro18Leu	VUS	PM2_supp
Missense	MYBPC3	NM_000256.3	c.50G>A	p.Arg17Gln	VUS	BP4
Missense	MYBPC3	NM_000256.3	c.2671C>T	p.Arg891Trp	VUS	PM2_supporting, PP3
Intronic affecting splicing	TTN	NM_003319.4	c.14829+5G>T		VUS	PM2_supporting, PP3, PS4_supp

Missense	RYR2	NM_001035.2	c.5021A>G	p.His1674Arg	VUS	PM2_supporting, PP3
Missense	TNNT2	NM_001001430.2	c.416G>T	p.Arg139Leu	VUS	PM2, PP3, PM5
Missense	PKP2	NM_001005242.3	c.2501C>T	p.S834F	VUS	PM2_supp
Missense	BAG3	NM_004281	c.337G>A	p.V113I	VUS	PM2_supp, BP4
Missense	FLNC	NM_001458.5	c.2411A>C	p.Lys804Thr	VUS	PM2_supp, PP3
Missense	FLNC	NM_001458.5	c.6451G>A	p.Gly2151Ser	VUS	PM2_supp, PP3, PS4_mod
Missense	DSG2	NM_001943	c.1013A>T	p.K338M	VUS	
Missense	DES	NM_001927	c.353G>A	p.Arg118His	VUS	PM2_supp, PP3
Missense	ACTC1	NM_005159	c.644A>G	p.K215R	VUS	PM2_supp, PP3
Missense	DSP	NM_001008844.3	c.859A>G	p.Asn287Asp	VUS	PM2_supp, PP3, PM5_supp

Missense	KLHL24	NM_017644.3	c.1525G>C	p.Gly509Arg	VUS	PM2_supp, PP3
Missense	GATA6	NM_005257.5	c.1106C>G	p.Pro369Arg	VUS	PM2_supp, PP3
Missense	GATA5	NM_080473.4	c.713G>A	p.Arg238His	VUS	PM2_supp, PP3
Delins	MYH7	NM_000257.4	c.5469_5471delinsATG	p.Asn1824Cys	VUS	PM2_supp
Missense	TNNI3	NM_000363.4	c.88G>A	p.Ala30Thr	VUS	PM2_supp, PP1
Missense	FLNC	NM_001458.5	c.1229T>C	p.Val410Ala	VUS	PM2_supp
Missense	TNNT2	NM_001001430.2	c.302A>G	p.Glu101Gly	VUS	PM2_supporting, PP3
Missense	MYH7	NM_000257.3	c.1351C>G	p.Gln451Glu	VUS	PM2, PM1, PS4_supp
Missense	PRKAG2	NM_016203.4	c.868A>G	p.Lys290Glu	VUS	PM2_supp, PP3, PM5_supp
Missense	LMNA	NM_170707.4	c.1070A>T	p.Asp357Val	VUS	PM2_supp, PM5, PP3

Missense	FLNC	NM_001458.5	c.6053G>C	p.Arg2018Pro	VUS	PM2_supp, PS4_supp
Missense	MYPN	NM_032578.4	c.3416G>A	p.Arg1139His	VUS	
Missense	TCAP	NM_003673.3	c.171C>G	p.C57W	VUS	PM2_supp, PP3
Missense	DES	NM_001927.4	c.1207G>C	p.Ala403Pro	VUS	PM2_supp, PP3
Missense	MYH6	NM_002471.4	c.3949C>A	p.Leu1317Ile	VUS	PM2_supp
Missense	TTN	NM_003319.4:c.13823C>T		p.Pro4608Leu	VUS	
Missense	DES	NM_001927	c.376G>A	p.Val126Met	VUS	PM2_supp, BS3_supp
Missense	MYPN	NM_032578.3	c.266A>G	p.Glu89Gly	VUS	PM2_supp, BP4
Missense	LAMA4	NM_001105206.2:c.2185C>A	NC_000006.11:g.112469527G>T	p.Gln729Lys	VUS	PM2_supp, BP4

Missense	FBN1	NM_000138.4:c.2926C>T	NC_000015.9:g.48782204G>A	p.Arg976Cys	VUS	PM2_supp, PM1, PP3
Missense	CALR3			p.Arg356His	VUS	PM2_supp, BP4
Missense	MYH7	NM_000257.3	c.2908G>A	p.Ala970Thr	VUS	PM2_supp
Missense	LDB3	NM_007078.2	c.1472T>A	p.Val491Glu	VUS	PM2_supp, BP4, PS4_supp
Missense	FLNC	NM_001458.4	c.3137C>T	p.Pro1046Leu	VUS	PM2_supporting
Splice-site (non-canonical intronic)	NRAP	NM_001261463.1	c.3915+5G>T		VUS	PM2_supporting
Missense	NRAP	001261463.1	c.4808G>A	p.Ser1603Asn	VUS	PM2_supporting, BP4
Missense	MYBPC3	NM_000256.3	c.151G>A	p.Ala51Thr	VUS	PM2_supporting, BP4
In-frame deletion	TTN	NM_003319.4	c.63569_63571del	p.Gly21190del	VUS	PM2_supp, PM4

Missense	JUP	NM_001352773	c.176C>A	p.Thr59Asn	VUS	PM2_supp, BP4
Missense	DSP	NM_001008844	c.2911G>A	p.G971R	VUS	PM2_supp
Missense	KLHL24	NM_017644.3	c.923C>T	p.Ser30Phe	VUS	PM2_supp
Missense	FLNC	NM_001458.5	c.1081C>T	p.Arg361Cys	VUS	sin criteiros
Missense	MYOT	NM_001135940	c.419T>C	p.Val140Ala	VUS	PM2_supp
Missense	FHOD3	NM_001281739.3	c.815C>T	p.Thr272Met	VUS	PM2_supp, PP3
Missense	SCN5A	NM_198056.2	c.2236G>A	p.Glu746Lys	VUS	
Missense	RYR2	NM_001035	c.11711G>A	p.3904R>Q	VUS	PM2_supp, PP3
Missense	MYBPC3		c.667G>A	p.E223K	VUS	PM2_supp, PP3
Missense	MYBPC3	NM_000256.3	c.50G>A	p.Arg403Gln	VUS	BP4

Missense	LMNA	NM_170707.3	c.238G>T	p.Ala80Ser	VUS	PM2_supp
Missense	SCN5A			p.Ser835Leu	VUS	PM2_supp, PP3
Missense	MYBPC3	NP_000247.2		p.Ala125Val	VUS	
Missense	MYH7	NM_000257.4	**c.5422G>A**	p.Gly1808Ser	VUS	
Missense	DSC2	NM_024422.6	c.421C>T	p.Pro141Ser	VUS	PM2_supp, PP3
Missense	EMD	NM_000117.2:c.277G>T	c.277G>T	p.Asp93Ty	VUS	
Missense	LAMA2	NM_000426.3	c.5146G>A	p.Gly1716Arg	VUS	
Splice-site (canonical ±1/±2)	TRIM63	NM_032588.3	c.597+1G>A		VUS	
Missense	MYBPC3	NM_000256	c.814C>T	p.272R>C	VUS	PM2_supporting
Missense	MYBPC3	NM_000256	c.3764C>G	p.1255A>G	VUS	PM2_supporting, PP3.
Missense	NEXN		Glu470del		VUS	PM2_supporting, PM4

Missense	MYBPC3	NM_000256.3	c.377A>G	p.Glu126Gly	VUS	
Missense	MYPN			Arg955Gln	VUS	PM2_supporting, BP4
Missense	MYH7	NM_000257.4	c.563C>T	p.Thr188Ile	VUS	PM2_supp, PP3
Sinonima	MYBPC3	NM_000256.3	c.2304C>A	p.(Val768=)	VUS+	PM2,_supp, PP3, PP1
Truncating - Frameshift	BAG3	NM_004281.4	c.1073_1076del	p.Pro358LeufsTer4	VUS+	PM2_supp, PVS1_Strong
Splice-site (canonical ±1/±2)	FLNC	NM_001458.4	c.6484+2T>A		VUS+	PM2_supp, PS4_supp, PVS1_mod
CNV (duplication)	PLN		NC_000006.11:g.(?_118869427)_(118880244_?)x4		VUS+	1A, 3A, 4H.

CNV (duplication)	PLN		NC_000006.11:g.(?_118869427)_(118880244_?)x4		VUS +	1A, 3A, 4H.
Missense	FKTN	NP_001073270.1	c.182T>G	Phe61Cys	VUS +	PM2_supp, PM3_supp, PP3
Missense	TNNT2	NM_001001430.2	c.452G>A	p.Arg151His	VUS +	PM2_supp, PM1, PS4_supp, PP3
Splice-site (canonical ±1/±2)	TTN	NM_003319.4	c.64076-1G>C		VUS +	PM2_supporting, PVS1_mod
Missense	TNNT2	NM_001001430.3	c.517C>G	p.Arg173Gly	VUS +	PM2, PS4_supp, PM5
Splice-site (canonical ±1/±2)	TTN	NM_003319.4	c.20074+1G>C		VUS +	PM2_supp, PVS1_mod

Abbreviations: ACMG/AMP = American College of Medical Genetics and Genomics and the Association for Molecular Pathology; LP = Likely Pathogenic; P = Pathogenic; VUS = Variant of Uncertain Significance.

Table S2. Missing data patterns in the survival analysis sample

Variable	N available	N missing	Missing (%)
Temporal cohort	445	3	0.7%
Age at transplantation	445	3	0.7%
Functional gene group	428	20	4.5%
Clinical risk group	406	42	9.4%

Data are shown for the final analytic samples after applying inclusion criteria (DCM, positive genetic test, exclusion of metabolic disease genotypes and DMD). Little's test for Missing Completely at Random (MCAR) was not significant ($\chi^2 = 3.96$, df = 9, p = 0.914), supporting the use of complete-case analysis.

Table S3. Baseline characteristics of included vs. excluded participants

Characteristic	Excluded (n=707)	Included (n=657)	p-value
Age at transplantation, years (mean ± SD)	52.5 ± 13.3	50.5 ± 13.7	0.007
Male sex, n (%)	511 (71.5%)	395 (67.9%)	0.16
DCM phenotype, n (%)	535 (74.5%)	409 (69.8%)	0.06
LVEF, % (mean ± SD)	26.1 ± 12.7	27.3 ± 12.9	0.07
Temporal cohort, n (%)			<0.001
- 2010-2014	273 (38.2%)	116 (19.9%)	
- 2015-2019	256 (35.8%)	233 (40.0%)	
- 2020-2023	186 (26.0%)	233 (40.0%)	

Supplemental Table Y compares baseline characteristics between participants included in the primary survival analysis (n = 586) and those excluded (n = 718). Included participants were slightly younger (p = 0.007), with no significant differences in sex, DCM phenotype, or LVEF. The temporal cohort distribution differed (p < 0.001), with more included participants in recent years, reflecting the retrospective nature of genetic testing.

Table S4. Baseline characteristics and outcomes of heart transplant recipients according to genetic testing results: GT-positive vs GT-negative.

Variable	GT+ (n=348)	GT- (n=309)	P value
Age at HTx, years	50.1 (12.2)	52.3 (12.8)	0.03
BMI, kg/m²	25.2 ± 4.1	25.7 ± 4.3	0.19
HTN, n (%)	62 (18%)	87 (28%)	<0.01
DLP, n (%)	85 (24%)	85 (28%)	0.34
DM on insulin, n (%)	36 (11%)	58 (19%)	<0.01
Creatinine, mg/dL	1.2 ± 0.6	1.3 ± 0.7	0.19
Bilirubin, mg/dL	1.2 ± 0.9	1.3 ± 1.3	0.27
Inotropes, n (%)	110 (32%)	107 (35%)	0.44
VAD pre-HTx, n (%)	78 (23%)	86 (28%)	0.12
mPAP, mmHg	27.7 ± 8.7	26.6 ± 8.7	0.15
PCP, mmHg	20.3 ± 7.4	19.3 ± 7.5	0.11
Cardiac index, L/min/m²	3.9 ± 1.1	4.1 ± 1.2	0.05
Transpulmonary Gradient, mmHg	7.4 ± 3.6	7.4 ± 3.6	0.93
Urgent listing, n (%)	109 (31%)	113 (36%)	0.16
Graft failure, n (%)	63 (18%)	46 (15%)	0.25
Htx waiting list, days	72 [18-221]	63 [14-182]	0.13
Need for post-op assistance, n (%)	47 (13%)	72 (23%)	<0.01
ICU admission days	6 [4–10]	6 [4–11]	0.38

Values are mean ± SD, n (%), or median [interquartile range].

Abbreviations: BMI = body mass index; DLP = dyslipidemia; DM = diabetes mellitus; GT = genetic testing; HTN = arterial hypertension; HTx = heart transplantation; ICU = intensive care unit; LP/P = likely pathogenic/pathogenic; mPAP = mean pulmonary artery pressure; PCP = pulmonary capillary pressure; VAD = ventricular assist device.

Continuous variables were compared using the Student's t-test or Mann-Whitney U test, as appropriate. Categorical variables were compared using the chi-square test or Fisher's exact test. A p-value <0.05 was considered statistically significant.

Table S5. Diagnostic Yield of Genetic Testing by Cardiomyopathy Phenotype and Cohort Type

Phenotype	Overall cohort, n (%)		Proband & NGS-tested cohort, n (%)	
DCM	448	207 (46)	409	170 (42)
HCM	136	93 (68)	120	80 (67)
ACM	39	22 (56)	39	22 (56)
RCM	34	25 (74)	18	10 (55)
Total	657	347 (53)	586	282 (48)

Diagnostic yield of comprehensive genetic testing in 657 heart transplant recipients with cardiomyopathy. Yield is defined as the identification of a pathogenic or likely pathogenic (P/LP) variant. The "Overall cohort" includes all tested patients. The "Proband & NGS-tested cohort" is restricted to index cases from each family who underwent next-generation sequencing (NGS), thereby excluding relatives identified via cascade family screening and probands who underwent only targeted sequencing for a familial variant or TTR gene evaluation. Abbreviations: ACM, arrhythmogenic cardiomyopathy; DCM, dilated cardiomyopathy; HCM, hypertrophic cardiomyopathy; RCM, restrictive cardiomyopathy.

Table S6. Genetic Findings in Heart Transplant Recipients with Positive Genetic Test Combining Cardiac Skeletal Muscle Phenotypes (n=14)

Gene	Number of Patients	Phenotype at Presentation
LMNA	3	DCM (2), ACM (1)
DMD	3	DCM (3)
MYH7	2	DCM (1) RCM (1)
FKTN	1	DCM (1)
EMD	1	DCM (1)
FHL1	1	HCM (1)
LAMP2	1	HCM (1)
MT-TL1	1	DCM (1)
MYBPC3	1	DCM (1)

Spectrum of pathogenic or likely pathogenic variants identified in 19 patients from the Genbase-Htx cohort who presented with features of both cardiomyopathy and skeletal myopathy. Phenotypes correspond to the primary morphological diagnosis of cardiomyopathy at presentation. Abbreviations: DCM, dilated cardiomyopathy; ACM, arrhythmogenic cardiomyopathy; HCM, hypertrophic cardiomyopathy; RCM, restrictive cardiomyopathy.

Table S7. Reclassification of Initial Clinical Etiology Following Genetic Testing in a Cohort of Heart Transplant Recipients

	Final Etiology		
Initial Etiology	Non-Genetic	Genetic	Total
Idiopathic	212	143	355
Familial / Genetic Cardiomyopathy	64	180	244
Presumed Acquired/"Second-Hit" Etiologies			42
Chemotherapy-Induced Cardiomyopathy	11	4	15
Myocarditis	10	5	15
Alcohol-Induced Cardiomyopathy	4	1	5
Tachycardia-Induced Cardiomyopathy	1	3	4
Peripartum Cardiomyopathy	1	2	3
Other	2	3	5
Neuromuscular Disease–Related Cardiomyopathy	0	4	4
Hypertensive	3	0	3
Inflammatory Cardiomyopathy	2	2	4
Total	310	347	657

Diagnostic reclassification of 657 heart transplant recipients based on comprehensive genetic testing. The "Initial Etiology" reflects the pre-test clinical diagnosis. The "Final Etiology"

indicates post-test classification based on the presence ("Genetic") or absence ("Non-Genetic") of a P/LP variant. Genetic testing reclassified 40% (143/355) of idiopathic cases and identified a genetic cause in 36% (15/42) of patients with a presumed acquired etiology.

Table S8. Distribution of Pathogenic Variants and Genetic Testing Positivity in Dilated Cardiomyopathy Cohorts

Gene	Escobar-López (n=1005)		Verdonschot (n=689)		Genbase-Htx (n=448)		
	n	%	n	%	n	%	CI
TTN	141	38.7	66	51.2	53	25.60	20.1-32.0
LMNA	31	8.5	21	16.28	26	12.56	8.7-17.8
FLNC	21	5.8	3	2.33	8	3.86	2.0-7.4
DSP	31	8.5	4	3.10	9	4.35	2.3-8.0
RBM20	20	5.5	5	3.9	5	2.42	1.0-5.7
BAG3	24	6.6	1	0.8	10	4.83	2.7-8.6
MYH7	17	4.7	7	5.43	10	4.83	2.7-8.6
MYBPC3	17	4.7		0.00	14	6.76	4.1-11.0
TNNT2	13	3.6	4	3.1	5	2.42	1.0-5.7
TPM1	5	1.4	5	3.9	1	0.48	0.1-2.7
ACTC1	2	0.6	2	1.6	—	0.00	—
EMD	1	0.3	1	0.8	32	15.46	11.2-21.0
PLN	2	0.6	3	2.3	3	1.45	0.4-4.2
TNNC1	2	0.6	1	0.8	—	0.00	—
TNNI3	4	1.1	—	0.00	—	0.00	—
TMEM43	1	0.3	—	0.00	—	0.00	—

PRDM16	1	0.3	—	0.00	—	0.00	—
SCN5A		—	1	0.8	—	0.00	—
LAMP2		—	—	0.00	4	1.93	0.7-5.1
NEXN		—	2	1.6	—	0.00	—
DMD	18	5.0	—	—	12	5.80	3.4-9.9
JPH2	1	0.3	—	—	—	—	—
SGCD	1	0.3	—	—	—	—	—
DES		—	—	—	1	0.48	0.1-2.7
DMPK		—	—	—	1	0.48	0.1-2.7
DSG2		—	—	—	1	0.48	0.1-2.7
FKTN		—	—	—	1	0.48	0.1-2.7
FHL1		—	—	—	1	0.48	0.1-2.7
GBE1		—	—	—	1	0.48	0.1-2.7
GYG1		—	—	—	1	0.48	0.1-2.7
MT-TL1		—	—	—	1	0.48	0.1-2.7
RRAGD		—	—	—	1	0.48	0.1-2.7
TBX20	3	0.8	—	—	1	0.48	0.1-2.7
TCAP		—	—	—	1	0.48	0.1-2.7
MYL2			1	0.8			
TRIM63		—	—	—	1	0.48	0.1-2.7

Positivity (%)	364	36	129	19	207	46	42-51

Comparative genetic landscape of three dilated cardiomyopathy (DCM) cohorts. Upper section: Distribution of pathogenic/likely pathogenic variants among genotype-positive patients, expressed as the percentage of patients with a variant in each specified gene. Bottom row: Overall genetic testing positivity rate, indicating the percentage of all patients in each cohort with an identifiable pathogenic variant. Cohorts: Escobar-López et al. (n=1005) and Verdonschot et al. (n=689) represent ambulatory DCM populations. The *Genbase-Htx* cohort (n=488) comprises patients with DCM who progressed to end-stage heart failure requiring transplantation.

Table S9. Comparison of Pathogenic Gene Variant Distribution Across Dilated Cardiomyopathy Cohorts: General vs. End-Stage Heart Failure Cohorts

Gene	Escobar-López (%)	Verdonschot (%)	Genbase-Htx (%)	p (Genbase vs Escobar)	p (Genbase vs Verdonschot)
TTN	35.48	51.16	25.60	<0.01	<0.01
LMNA	9.75	16.28	12.56	0.09	0.08
FLNC	5.85	2.33	3.86	0.11	0.13
DSP	8.19	3.10	4.35	<0.01	0.25
RBM20	7.80	3.88	2.42	<0.01	0.16
BAG3	6.43	0.78	4.83	0.22	<0.01
MYH7	5.65	5.43	4.83	0.53	0.65
MYBPC3	3.70	0.00	6.76	<0.01	<0.01
EMD	0.39	0.78	15.46	<0.01	<0.01
Positivity	37	19	46	<0.01	<0.01

Gene-specific frequencies of pathogenic or likely pathogenic variants in three dilated cardiomyopathy cohorts: Escobar-Lopez et al. (n=1005), Verdonschot et al. (n=689) -both ambulatory DCM cohorts-, and the current Genbase-Htx cohort (n=488) -DCM requiring transplantation-. P-values from chi-square or Fisher's exact tests are shown for genes with a frequency ≥2% in at least

one cohort. No adjustment for multiple testing was applied; these comparative results should be considered exploratory.

Table S10. Distribution and comparison of sarcomeric and non-sarcomeric gene variants in ambulatory versus end-stage HCM cohorts

Gene	Alfares (%)	Walsh (%)	Genbase-HTx (%)	p (Genbase vs Alfares)	p (Genbase vs Walsh)
MYBPC3	33.0	34.6	33.0	1.00	0.62
MYH7	27.7	25.6	28.0	0.94	0.42
TNNT2	6.4	6.4	6.5	1.00	1.00
TTR	5.3	3.9	5.0	0.92	0.43
ACTC1	4.3	5.1	4.3	1.00	0.63
PRKAG2	4.3	3.9	3.2	0.44	0.64
LAMP2	2.1	2.6	3.2	0.33	0.65
TNNI3	3.2	3.9	3.2	1.00	0.68
TPM1	3.2	3.9	4.3	0.46	0.87
FHOD3	2.1	—	2.2	—	—
ALPK3	1.1	1.3	1.1	—	—
FHL1	—	—	1.1	—	—
MT-WT	1.1	1.3	1.1	—	—
MYL2	1.1	1.3	1.1	—	—

MYL3	1.1	1.3	1.1	—	—
TRIM63	1.1	1.3	1.1	—	—
Positivity	32.0	32.0	68.0	<0.01	<0.01

Comparative genetic landscape of three hypertrophic cardiomyopathy (HCM) cohorts. Upper section: Distribution of pathogenic/likely pathogenic variants among genotype-positive patients, expressed as the percentage of patients with a variant in each specified gene. Bottom row: Overall genetic testing positivity rate, indicating the percentage of all patients in each cohort with an identifiable pathogenic variant. Dashes (—) indicate that the gene was not reported or no variants were identified in that cohort. Cohorts: Alfares et al. (n=2912) and Walsh et al. (n=3267) represent ambulatory HCM populations. The *Genbase-Htx* cohort (n=136) comprises patients with HCM who progressed to end-stage heart failure requiring transplantation. P-values from chi-square or Fisher's exact tests are shown for genes with a frequency ≥2% in at least one cohort. No adjustment for multiple testing was applied; these comparative results should be considered exploratory.

Table S11. Distribution and comparison gene variants in ambulatory versus end-stage ACM cohorts

Gene	Genbase (%)	Bariani et al. (%)	p-value
DSG2	27.3	11.1	0.03
PKP2	27.3	37.8	0.63
DSP	9.0	37.8	0.01
LMNA	9.0	0.9	0.04
TMEM43	9.0	0.46	<0.01
DES	4.6	2.3	0.41
DSC2	4.6	0.9	0.23
TNNI3	4.6	0	0.08
FLNC	0	7.8	0.37
Positivity	56.4	49.1	0.41

Comparative genetic landscape of two arrhythmogenic cardiomyopathy (ACM) cohorts. Upper section: Distribution of P/LP variants among genotype-positive patients, expressed as the percentage of patients with a variant in each specified gene. Bottom row: Overall genetic testing positivity rate, indicating the percentage of all patients in each cohort with an identifiable pathogenic variant. Cohorts: Bariani et al. (n=446) represents an

ambulatory ACM population. The Genbase-Htx cohort (n=39) comprises patients with ACM who progressed to ESHF requiring HTx. P-values from chi-square or Fisher's exact tests are shown for genes with a frequency ≥2% in at least one cohort.

Table S12. Distribution and comparison of gene variants in ambulatory versus end-stage RCM cohorts

Gene	Gallego-Delgado et al.	GenbaseHTx
TTR	–	48.0
FLNC	9.4	8.0
MYH7	12.5	8.0
DES	9.4	8.0
TNNT2	3.1	8.0
EMD	–	4.0
FHL1	–	4.0
GLA	–	4.0
LAMP2	3.1	4.0
MYBPC3	6.3	4.0
LMNA	6.3	–
TCAP	3.1	–
TNNI3	3.1	–
TPM1	3.1	–
Positivity	59.4	85.3

Comparative genetic landscape of two restrictive cardiomyopathy (RCM) cohorts. Upper section: Distribution of P/LP variants among

genotype-positive patients, expressed as the percentage of patients with a variant in each specified gene. Bottom row: Overall genetic testing positivity rate, indicating the number and percentage of all patients in each cohort with an identifiable pathogenic variant. Dashes (—) indicate that the gene was not reported or no variants were identified in that cohort. Cohorts: Gallego-Delgado et al. (n=32) represents an ambulatory RCM population. The Genbase-HTx cohort (n=34) comprises patients with RCM who progressed to ESHF requiring HTx. P-values from chi-square or Fisher's exact tests are shown for genes with a frequency ≥2% in at least one cohort.

Table S13. Heart Transplant Recipients Carrying Multiple Pathogenic or Likely Pathogenic Variants

Patient ID	Phenotype	Gene 1	c.DNA/ Protein 1	P	Gene 2	c.DNA/ Protein 2	P
**43**	DCM	*RBM20*	p.Arg634Gln	P	*TTN*	p.Glu596Lysfs*21	P
**510**	HCM	*MYBPC3*	p.Arg1022Pro	LP	*FLNC*	p.Pro2308Leu	LP
**545**	DCM	*MYBPC3*	c.2603-2A>G	P	*MYBPC3*	p.Glu441Lys	**RF**
**1035**	DCM	*PKP2*	p.Glu259Glyfs*77	P	*DSG2*	p.Thr335Ala	P
**1054**	DCM	*DSP*	p.Gln1667Hisfs*14	LP	*MYL3*	p.Ala57Asp	LP
**1058**	DCM	*TTN*	p.Trp16080*	P	*MYH7*	p.Arg1781His	LP
**1178**	DCM	*TTN*	p.Arg8276*	P	*MYBPC3*	p.Arg495Gly	P
**48**	HCM	*TRIM63*	p.Gln53*	LP	*TRIM63*	p.Ser161Cysfs*8	P
**139**	DCM	*TTN*	c.27616+1G>A	LP	*TTN*	c.47107+1G>A	LP
**760**	DCM	*DSG2*	c.-1_1delGAinsCG	LP	*DSG2*	c.523+4A>G	LP
**1051**	DCM	*MYBPC3*	p.Leu224del	LP	*MYBPC3*	p.Thr227Ile	LP
**1262**	RCM	*TNNT2*	p.Arg94Leu	LP	*MYL2*	-	LP
**1289**	RCM	*TNNT2*	p.Arg94Leu	LP	*MYL2*	-	LP
**583**	DCM	*FKTN**	p.Gly424Ser	LP	-	-	-
**1113**	DCM	*HJV**	p.Gly320Val	LP	-	-	-
**1228**	DCM	*DES**	p.Arg16Cys	LP	-	-	-

Values represent findings for patients with complex genetic architectures. For patients with digenic or compound heterozygous inheritance, variants in both Gene 1 and Gene 2 are provided.

Abbreviations: ACM = arrhythmogenic cardiomyopathy; DCM = dilated cardiomyopathy; HCM = hypertrophic cardiomyopathy; HTx = heart transplantation; LP = likely pathogenic; P = pathogenic; RCM = restrictive cardiomyopathy; RF = risk factor.

* Denotes homozygous status. All other entries represent either digenic inheritance (variants in two different genes) or compound heterozygous inheritance (two different variants in the same gene). RF (Risk Factor) indicates a variant of intermediate effect that contributes to the clinical phenotype in an oligogenic model but does not independently fulfill ACMG criteria for LP/P status. Full variant nomenclature (including transcript IDs) and specific ACMG criteria supporting each classification are provided in Supplemental Table 3.

Table S14. Summary of Prior Studies Assessing the Diagnostic Yield of Genetic Testing in Heart Transplant Recipients With Cardiomyopathy

Title	Year	Sample size (n)	Study design	Genetic testing method	GT reporting	Key results
GT Practices and Pathological Assessments in ESHF Patients Undergoing HTx and LVAD	2024	196	Retrospective, single-center, 2018–2023, HTx/LVAD, NICM, ICM, mixed (CM out of proportion to CAD) probands?	No information (NGS? Sanger? Which panels? CNV or structural variants?)	No ACMG criteria provided	GT rate: 54%; NICM/mixed N=196, 70 (36%) positive. Most frequent: *TTN* (32%), *MYBPC3* (13%), *TTR* (11%). GT yield: ACM 75% (3/4), HCM 69% (9/13), RCM 48% (10/21), DCM 30% (42/138), NDLVC 30% (6/20), p=0.02. Multiple LP/P variants in 9% (6/70). 22% GT+ in mixed phenotype. Family history associated with higher yield.
Diagnostic yield of GT in HTx recipients	2022	45	Single-center 1995–2020, HTx, active follow-up, probands?	NGS CM-panel (36, 51, or 59 genes depending on year).	ACMG criteria mentioned in methods	5 pre-HTx GT, 31 post-HTx GT. GT+ in NICM: 12/31 (39%). Most frequent: *TTN* 7/31, *MYBPC3* 2/31. Similar diagnostic yield

with prior CM				CNV/structural variants?	but not provided	regardless of family history or second hits.
Genetic Contribution to End-Stage Cardiomyopathy Requiring Heart Transplantation	2023	122 (101 adults + 21 pediatrics)	2011–2019, Canada, single-center, probands?	WES/WGS of explanted hearts; mitochondrial sequences analyzed; parvovirus B19 sequences; CNV analysis; no ACMG criteria	Not provided	44% positive (n=54). DCM 42%: *TTN* 42.9%, *LMNA* 16.7%, *BAG3* 7.1%, *DSP* 7.1%. HCM 18 patients (50%+): *MYBPC3* (5 variants), *MYH7* (2), *TNNT2* (1). ACM=3, RCM=2 (pediatrics). WES/WGS identified 3 large structural variants + 2 noncanonical splicing variants (4% of cohort). Parvovirus enrichment in 2 tissues. Distribution of variants similar across cohorts.
Genetic basis of familial DCM patients undergoing heart	2016	52	3 HTx centers, Spain, familial DCM	NGS 59 gene panel	Not specified	EG+ in 40% initially, after cosegregation EG+ 73%. Most frequent: *EMD (13), TTN (10), BAG3 (4), LMNA (3), FLNC (2), TNNT2 (2), DMD, DSC2, DSP, MYH7, PKP2, ABCC9, TPM1.*

transplantati on						Excluding *EMD* c.77T>C founder mutation, 23.1% had multiple genetic variants.

This table summarizes key studies published prior to the present work. All listed studies are retrospective, single-center, and focus on probands with end-stage cardiomyopathy.

Abbreviations: ACM, arrhythmogenic cardiomyopathy; ACMG, American College of Medical Genetics and Genomics; CNV, copy number variant; DCM, dilated cardiomyopathy; ESHF, end-stage heart failure; GT, genetic testing; HCM, hypertrophic cardiomyopathy; HTx, heart transplantation; ICM, ischemic cardiomyopathy; LP/P, likely pathogenic / pathogenic; LVAD, left ventricular assist device; NDLVC, non-dilated left ventricular cardiomyopathy; NGS, next-generation sequencing; NICM, non-ischemic cardiomyopathy; RCM, restrictive cardiomyopathy; WES, whole-exome sequencing; WGS, whole-genome sequencing.
